# Modelling the dynamics of polar auxin transport in inflorescence stems of *Arabidopsis thaliana*

**DOI:** 10.1093/jxb/erv471

**Published:** 2015-11-02

**Authors:** Kees J.M. Boot, Sander C. Hille, Kees R. Libbenga, Lambertus A. Peletier, Paulina C. van Spronsen, Bert van Duijn, Remko Offringa

**Affiliations:** ^1^Plant Biodynamics Laboratory, Institute of Biology Leiden, Leiden University, 2333 EB Leiden, The Netherlands; ^2^Department of Molecular and Developmental Genetics, Institute of Biology Leiden, Leiden University, 2333 EB Leiden, The Netherlands; ^3^Mathematical Institute, Leiden University, 2333CA, Leiden, The Netherlands; ^4^Fytagoras, 2333 EB Leiden, The Netherlands

**Keywords:** Arabidopsis, chemiosmotic theory, IAA, modelling, mutants, polar auxin transport.

## Abstract

The polar transport of the plant hormone auxin has been the subject of many studies, several involving mathematical modelling. Unfortunately, most of these models have not been experimentally verified. Here we present experimental measurements of long-distance polar auxin transport (PAT) in segments of inflorescence stems of *Arabidopsis thaliana* together with a descriptive mathematical model that was developed from these data. It is based on a general advection–diffusion equation for auxin density, as suggested by the chemiosmotic theory, but is extended to incorporate both immobilization of auxin and exchange with the surrounding tissue of cells involved in PAT, in order to account for crucial observations. We found that development of the present model assisted effectively in the analysis of experimental observations. As an example, we discuss the analysis of a quadruple mutant for all four *AUX1/LAX1–LAX3* influx carriers genes. We found a drastic change in the parameters governing the exchange of PAT channels with the surrounding tissue, whereas the velocity was still of the order of magnitude of the wild type. In addition, the steady-state flux of auxin through the PAT system of the mutant did not exhibit a saturable component, as we found for the wild type, suggesting that the import carriers are responsible for the saturable component in the wild type. In the accompanying Supplementary data available at *JXB* online, we describe in more detail the data-driven development of the model, review and derive predictions from a mathematical model of the chemiosmotic theory, and explore relationships between parameters in our model and processes and parameters at the cellular level.

## Highlight

An experimental and mathematical approach to polar auxin transport results in a model based on an extended general advection–diffusion equation including auxin immobilization and surrounding tissue exchange that accounts for crucial observations.

## Introduction

Auxin is a plant hormone endowed with a unique transport system. Interest in auxin transport dates back to 1880, when Charles Darwin reported on a transmissible signal that is involved in the phototropy of Canary grass coleoptiles (Darwin and Darwin, 1880). Later this signal was identified as indole-3-acetic acid (IAA) (Went, 1928). More than a century of research has shown that auxin is a key chemical player in almost every aspect of plant growth and development. At present auxin may even be regarded as a plant equivalent of animal morphogens, conferring positional information in a concentration-dependent manner in pattern formation, such as, for example, phyllotaxis (Friml, 2003; Bainbridge *et al.*, 2008).

Critical in auxin’s role as a ubiquitous chemical messenger is both a short- and long-range polar cell–cell transport (Berleth and Sachs, 2001). Long-range transport (centimetres and more) is the experimentally most accessible part of the polar auxin transport (PAT) system and, not accidentally, it was the first that was discovered and subsequently extensively studied. Since the 1960s, when radioactive auxin became available, the following overall picture emerged: auxin, which is synthesized in apical shoot meristems and leaf primordia, is transported in a basal direction by specialized cells that reside in the vascular bundles of leaves and of vegetative and generative (inflorescence) stems. In the root, auxin is transported in the central cylinder towards the root tip, where the transport direction is reversed and auxin is transported over a relatively short distance in an upward direction by root cap and epidermal cells. For a more detailed overview of earlier and recent work on long-range auxin transport, the reader is referred to Friml and Palme (2002).

With the advent of molecular genetics, the main focus of PAT research shifted towards a search for genes and their protein products involved in PAT. According to the current model the transport direction is governed by apical, basal, or sometimes lateral plasma membrane (PM) localization of members of the PIN-FORMED (PIN) auxin transport proteins, a class of putative auxin anion carriers/channels, as assumed in the chemiosmotic theory. Another class of proteins consists of the P-GLYCOPROTEIN, MULTIDRUG RESISTANCE, and ATP-BINDING CASSETTE SUBFAMILY B (PGP, MDR, and ABCB) auxin transport proteins, whose distribution is non-polar. ABCBs have been proposed to regulate the amount of auxin in the cell available for PAT. A third class of auxin transport proteins belongs to the AUX1/LIKE-AUX1 (AUX1/LAX) family of influx carriers/channels. In Arabidopsis, this family consists of four genes: *AUX1*, *LAX1*, *LAX2*, and *LAX3*, which are all non-polarly localized in the PM. It is assumed that they play a role in keeping IAA in the transporting cells, thereby overcoming leakage of auxin from the transport channels (Kramer, 2004; Geisler and Murphy, 2006; Krecek *et al.*, 2009; Petrasek and Friml, 2009; Peret *et al.*, 2012).

In short, over the past few decades tremendous progress has been made in revealing the genes and their protein products involved in PAT, and we observed an increasing number of computational models aimed at understanding the role of the distribution of auxin in time and space, during growth and developmental processes. These models rely largely on observed expression patterns of PAT genes instead of direct measurements of auxin fluxes (cf. Kramer, 2008). The problem with these correlation studies is that it has still not been resolved in mechanistic terms how PAT proteins, for example members of the PIN family, might facilitate PAT (Luschnig and Vert, 2014). As a first step in contributing to solve this problem, we decided to develop an experimental system, satisfying the following requirements: (i) the system must allow direct measurements of PAT and must be derived from model plants with a variety of accessible putative PAT mutants, such as Arabidopsis; and (ii) there should be some theoretical framework to begin with for interpreting and evaluating real data.

In order to satisfy the first requirement, we adopted, with some essential modifications, the classical donor–receiver assay, using inflorescence stem segments from *Arabidopsis thaliana*. To date, the classical donor–receiver assays are still the preferred way to measure directly average mass flows of (radioactive) auxin through the system (i.e. at the macroscopic level), thus providing reliable primary information as to the dynamics of PAT.

The second requirement confronted us with a lack of a suitable macroscopic mathematical model of PAT, namely at the level of a stem segment. There is theory though, the most popular being the chemiosmotic theory. It describes intercellular auxin transport through an array of PAT cells by transmembrane diffusion, where specific export carriers placed polarly in the membrane at one side of the cell are responsible for the asymmetry or polarity of the fluxes, and where the driving force of the polar transport component is the proton-motive force (cf. Rubery and Sheldrake, 1974; Raven, 1975).

Furthermore, the transport of intracellular auxin in PAT cells is assumed to be by simple diffusion through the cytoplast; that is, it is not by directed intracellular transport mechanisms such as, for example, cytoplasmic streaming or vesicle transport.

Although these two assumptions are the simplest to make, there is no direct experimental evidence for either. The mathematical model of the dynamics of this system identifies individual PAT cells. As this description is in between a macroscopic model that ignores cellular structure and a microscopic model that describes transport processes at the molecular scale, we call it a mesoscopic model for PAT. It does not cover either exchange of auxin with the tissue surrounding PAT cells or immobilization of auxin or other interferences. This class of models was derived by Mitchison (1980), Goldsmith, Goldsmith, and Martin (1981), which, we will call the MGGM model, following Kramer (2002).

The MGGM model suggests that PAT may effectively be described by a single advection–diffusion equation for the total auxin density in a continuum approximation at the macroscopic level (Kramer, 2008). The macroscopic effective diffusion coefficient and advection velocity have been related to mesoscopic parameters in the MGGM model heuristically (cf. Kramer, 2008 and Mitchison, 1980). An attempt to obtain a mathematically precise underpinning for this relationship was made by Chavarria-Krauser and Ptashnyk (2010), using so-called homogenization techniques. They took the anatomical structure of stem tissue and detailed microscopic cellular processes into account. However, as they observed themselves, they did not use the appropriate boundary conditions.

In our research we took the single macroscopic advection–diffusion equation suggested by the MGGM model as the starting point for interpreting real PAT data, thus validating this suggestion and extending initial trials by, for example, Hasenstein and Kaldewey (1983) and Hasenstein (1987). It turned out to be too simple to account for crucial observations made in our experimental PAT assays. Therefore, we gradually extended the model by subsequently combining modelling and fitting data by conducting further experiments. We found it necessary to account for, in particular, immobilization and leakage of auxin from the PAT channels.

Here we describe the ultimate model that we obtained, and demonstrate by its predictive potential its relevance for interpreting direct transport data. In addition, we provide our modelling decisions, motivated by experimental results and simulation. These have been documented in further detail in the Supplementary data available at *JXB* online. In particular, experiments with mutant Arabidopsis stems in which all four genes encoding the AUX1/LAX1–LAX3 influx carriers were knocked out (Bainbridge *et al.*, 2008) were crucial to arrive at our final model. The results of these experiments are presented herein. A follow-up paper is in preparation in which we describe screening of a substantial number of putative PAT mutants of Arabidopsis and where we use our ultimate model to interpret the data, also with the objective to refine the model further if the data require doing so.

The mathematical derivation of a detailed quantitative correspondence between macroscopic parameters in the model and the anatomical structure of stem tissues and microscopic cellular processes, although not straightforward, is a topic of further combined mathematical–experimental research. We will briefly comment on this problem in the Discussion, in particular with respect to the assumptions made by the MGGM model.

## Materials and methods

### Plant material and growth conditions

For all experiments, *A. thaliana* ecotype Columbia (Col-0) was used. Plants were grown on a mixture of 9:1 substrate soil and sand (Holland Potgrond) at 21 °C, a 16h photoperiod, and 70% relative humidity. Seeds from triple and quadruple mutants for the *AUX1/LAX* genes and the *AUX1-YFP* and *LAX3-GFP* reporter lines were provided by M. Bennett. The seeds from *DR5::GFP*, *PIN1-GFP*, *PIN-3-GFP*, and *PIN-7-GFP* reporter lines were obtained from J. Friml.

### Polar auxin transport measurements (standard PAT)

From 6-, 7-, and 8-week-old plants, 16mm long internodal segments were cut from the most basal part of the main stem of the inflorescences. Petri dishes were filled with molten paraffin. In the solid paraffin in each Petri dish, nine parallel 16mm long grooves between two rectangular wells (donor and receiver well, volume 2–3ml) or between one common donor well and nine individual receiver wells were cut from the paraffin. Stem segments, aligned in the original *in situ* orientation, were placed in the grooves. Subsequently these grooves were properly sealed by embedding the internodes in a small amount of silicon grease, covered by a small amount of grafting wax, thus obtaining a water-tight seal. The donor and receiver wells were filled with MA medium supplemented with 10mM MES (pH 4.8). Routinely, the donor wells contained 10^−4^ mol m^–3^ (10^−7^ M) ^3^H-labelled IAA (3-[5(*n*)-^3^H]IAA, specific activity 25 Ci mmol^–1^; Scopus Research BV, Veenendaal, The Netherlands).

The Petri dishes were placed on metal plates kept at 20 °C. At regular time intervals, receiver wells were emptied and replaced by fresh medium. Radioactivity of the samples was measured in an LKB liquid scintillation counter. For determining tissue profiles, segments were cut into 1, 2, or 4mm long pieces and transferred to scintillation liquid.

### Extended polar auxin transport measurements (extended PAT)

After an initial incubation time of 300min, the auxin-containing medium in the donor well was replaced by plain MA medium, whereupon the measured efflux profiles (0–600min) and the corresponding tissue profiles at 600min were analysed. The extended PAT assay does not allow measurement of tissue profiles at the end of the 300min initial incubation time; that is, the segments have to be kept intact for the second part of the experiment.

However, we were able to estimate the total amount of auxin present in the stem segments at 300min by adding the amount released into the donor between 300min and 600min, the amount released into the receiver between 300min and 600min, and the amount recovered in the stem segments after 600min. This amount should equal the amount predicted by the model with parameter values from the simulation of the transport profiles. As a rule, we only accepted deviations of a few percent.

### Microscopy

For light microscopy, segments from the basal part of inflorescences of Arabidopsis ecotype Col-0 were cut from the plant and either kept in 70% ethanol or used directly. Transverse sections were made with a bench-top microtome at a thickness of 50–70 μm, mounted on a glass slide in water. The sections were then examined with a Zeiss Axioplan Imaging upright light microscope (Carl Zeiss, Oberkochen, Germany.), equipped with a Zeiss Axiocam MRC 5 digital camera. Images were recorded using Zeiss Axiovision software and processed with Adobe Photoshop CS2 software (San Jose, CA, USA). Sections were also used to determine the anatomical parameters *S* (the cross-sectional area of a stem segment) and *S*_vb_ (sum of cross-sectional areas of vascular bundles). By cutting and weighing images of the sections, the percentage of *S*_vb_ per stem segment was determined.

For confocal scanning laser microscopy (CSLM), transverse sections at a thickness of 100 μm were made as described above. Longitudinal sections were made by cutting the stem by hand with a razor blade. Fresh sections were examined using a Zeiss Axioplan upright microscope equipped with a Biorad scanhead MRC 1024 ES (Hercules, CA, USA). The green fluorescent protein (GFP) and yellow fluorescent protein (YFP) fluorescence was detected using a gas krypton–argon laser at an excitation of 488nm and an emission of 522nm with a d.f. 32 filter. Digital images of CSLM optical sections were obtained using commercially available Lasersharp 2000 Biorad software and further processed using ImageJ version 1.38f (W. Rasband, USA) and Adobe Photoshop software.

#### Visualization of auxin transport 

Auxin localization in inflorescence stem segments was visualized in experiments with [2-^14^C]IAA (specific activity 55 mCi mmol^–1^, ARC, St Louis, MO, USA) using the Biomolex 700 Double-sided Silicon Strip Detector (Oslo, Norway). After a standard donor–receiver transport assay, stem segments that were cut lengthwise were placed on a glass slide and exposed overnight in the Bio-Molex 700 Imager. Data were analysed using the Biosplit software program.

### TLC analysis

Arabidopsis inflorescence stem segments were ground in liquid N_2_ and extracted with ethanol. Samples were spotted on thin-layer chromatography (TLC) silica gel60 F254 fluorescent plates and separated in a solvent containing *n*-hexane:ethylacetate:isopropanol:acetic acid (40:20:5:1, v/v/v/v) (Chung *et al.*, 2003). After running, the TLC plate was divided in 8–9 sections of 1.5cm, which were scraped off the plate, dissolved in ethanol, added to liquid scintillation vials, and counted in an LKB liquid scintillation counter.

### Mathematical modelling

A family of phenomenological mathematical models for the macroscopic dynamics of PAT was developed. In each model, the microscopic, biochemical, and physical details of transport at the cellular level were abstracted into a system of effective partial differential equations of reaction–diffusion–advection type to ensure a minimal number of required parameters while a good fit to experimental data for physiologically reasonable parameter values is maintained. Model complexity was increased when lack of proper fit or issues with the biological interpretation of changes in parameter values required us to do so (see the Supplementary data at *JXB* online for a more detailed account of the development of the model family).

### Numerical simulation and data fitting

Numerical simulation of the partial differential equations was performed in the COMSOL Multiphysics 4.2a finite element package (version 4.2.1.110) using the generalized-alpha time-dependent solver. For adequate resolution of the dynamics near the boundaries, the finite-element mesh was manually refined at both boundaries. As linear solver we used Direct1 (PARDISO). Computations ran in COMSOL Server 4.2a, coupled to MATLAB R2008b (version 7.7.0.471) through the ‘LiveLink for MATLAB’ interface.

Parameter optimization algorithms were implemented in MATLAB, using COMSOL as solver for the system of partial differential equations. As measure of the quality of fit, we used a cost function that is the sum of a transport and a profile part. The transport part is a weighted sum of the squared differences between simulation and the observed amount of auxin that accumulated in the receiver well at data time points. The profile part is an average squared difference between simulation and observed total amount of auxin in the 4mm long subsegments used in the determination of tissue profiles at the end of an experiment.

Optimization used the Gradient Descent Method, where the step size is determined by linear search in the negative gradient direction to increase the rate of convergence. The Golden Ratio method was used to find an approximation of a minimum of the cost function during the linear search. Amounts of tritium-labelled auxin could be measured up to an accuracy of ~1.5–2fmol. Therefore, optimization was stopped when the value of the cost function was less than this accuracy squared. The resulting fit we call optimal. Further details are provided in the Supplementary data at *JXB* online.

Physiological reasonable ranges for parameters were determined using literature data, direct measurements, or by approximation of parameters through dedicated computations (see the Supplementary data at *JXB* online). An initial setting of parameter values for optimization was obtained by manually tuning the parameters, using visual inspection of fit, while making sure that all parameter values remained in a physiologically reasonable range. This prevented automated optimization from ending in a local minimum of the cost functions, which is suboptimal.

## Results

### General transport characteristics of standard PAT

**Fig. 1.**
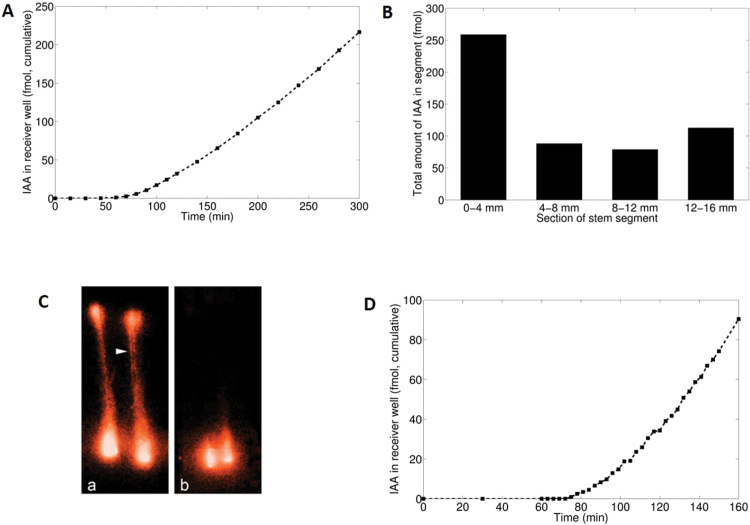
Data of transport profiles from the standard PAT. (A) Efflux profile. (B) Corresponding tissue profile obtained after 300min. (C) Visualization of tissue profiles obtained from a different experiment using [^14^C]IAA and imaging with the Bio-Molex 700 Imager: (a) polar orientation, (b) reverse orientation. The arrowhead indicates a likely vascular bundle. (D) Detail of an efflux profile from a separate experiment showing a transient obtained by frequent sampling (3min intervals). The results were obtained using the common receiver well. Consequently, the data from nine segments are averages, which are expressed per segment.

The transport characteristics shown in Fig. 1, though with some quantitative variation, represent the highly reproducible results from standard PAT, as described in the Materials and methods. Figure 1A shows an efflux profile, which we define as the accumulation of [^3^H]IAA in the receiver well as a function of time. Figure 1B shows the corresponding tissue profile, which we define as the distribution of [^3^H]IAA along the stem segments between the donor and receiver ends after a fixed incubation time, usually 300min. These profiles are collectively called transport profiles.

The efflux profile in Fig. 1A shows a time lag (in this case ~60min), followed by a transient (see also Fig. 1D), after which the efflux reaches a steady state (in this case ~1.1fmol min^–1^ per segment). The steady state was stable during a 10h experiment (not shown). Addition of the auxin transport inhibitor naphthylphthalamic acid (NPA) partly or completely blocked transport (not shown). The corresponding tissue profile (Fig. 1B) shows a relatively substantial accumulation of [^3^H]IAA at both the donor (0–4mm) and the receiver end (12–16mm) of the segments, whereas in the middle parts (4–8mm and 8–12mm) the amounts are about the same.

Using the Bio-Molex 700 Imager, we were able to visualize tissue profiles (Fig. 1C). In this experiment we had to use ^14^C-labelled IAA with a donor concentration of 1×10^–5^ M to obtain a sufficiently strong signal. After an incubation time of 330min, the segments were cut lengthwise and the two halves thus obtained were exposed overnight in the Bio-Molex reader. The display in Fig. 1C nicely shows that in the reverse orientation (basal part of the segment facing the donor well) auxin still accumulates at the donor end, but without further transport into the remaining part of the tissue.

The reproducibility of the standard PAT results from quite an extensive series of preliminary experiments in which we studied a number of experimental variables such as: age of plants, influence of part of the inflorescence from which the segments were taken, influence of pre-incubation either with or without 1×10^–7^ M unlabelled IAA, influence of NPA, and influence of incubation time. In addition, we checked conservation of radioactivity (mass balance satisfied loss of radioactivity from the donor well). From these preliminary experiments, we concluded that PAT measurements with basal stem segments of 6- to 7-week-old plants treated with 1×10^–7^M [^3^H]IAA were best suited for our experiments.

### Anatomy

It is a widely accepted paradigm that long-range PAT results from linear arrays of specialized cells (transport channels) that run over substantial distances through the stem and roots. At present there is substantial evidence that these cells are equipped with asymmetrically distributed PM-specific auxin–anion efflux carriers/channels and homogeneously distributed PM-localized influx carriers/channels. In general, detailed 3D reconstructions of the anatomical context of PAT channels are lacking. However, during development of the model we needed an anatomical context, albeit reduced to its assumed essentials, in order to be able to interpret real experimental data.

Therefore, to identify putative PAT cells in transverse and longitudinal cross-sections of inflorescence stem segments, the expression of the *PIN* and *AUX1/LAX* gene family members was checked using GFP- or YFP-based reporter lines. In addition we studied the expression of *DR5-GFP*, which provides information about cells that either have a relatively high IAA content or are more sensitive towards IAA.

**Fig. 2.**
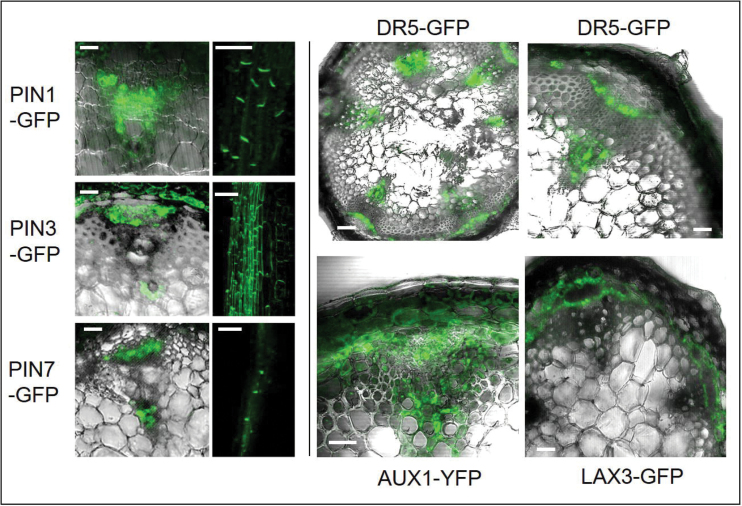
Expression patterns of PAT molecular markers. Expression of *PIN1-GFP*, *PIN1-3-GFP*, *PIN1-7-GFP*, *AUX1-YFP*, *LAX3-GFP*, and *DR5::GFP* promoter reporter fusions was observed in cells within both the phloem and xylem of vascular bundles. *PIN1-GFP*, and *PIN-7-GFP* were found to be mainly basally expressed, while *PIN3-GFP* expression was also observed at lateral cell membranes. Scale bar=50 μm.

Figure 2 shows a summary of the observed expression patterns. From these patterns we conclude that the best candidate PAT cells are paratracheal parenchyma cells in the xylem and small bundles of parenchyma cells in the phloem. The data do not provide information, however, of whether both cell types contribute equally to PAT or one of them is dominant. This may also depend on the developmental stage of the inflorescence stems.

### Model development

The ultimate mathematical model for long-range PAT that is presented herein resulted from step-by-step development that started from a model with the simplest assumptions and mathematical expressions, while in each step a minimal amount of complexity was added to the model when model predictions did not comply with experimental data. New experiments were performed to validate the extended model after each step. This section provides a concise account of this development. Further details are given in the Supplementary data at *JXB* online.

**Fig. 3.**
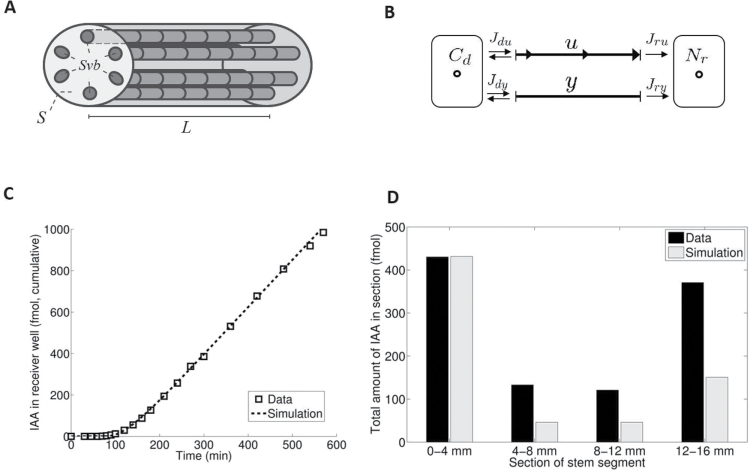
Fit with simple model. (A,B) Schematic representation of the (*u*,*y*) model in an inflorescence stem segment during PAT. *C*_d_, concentration of auxin in the donor well; *N*_r_, fmoles of auxin in the receiver well. *u* denotes the PAT compartment. *S*, cross-sectional area of the stem; *S*_vb_, sum of the cross-sectional areas of vascular bundles. For further details see the text. (C) Efflux profile of standard PAT and (D) corresponding tissue profile after 600min simulated with the (*u*,*y*) model. In our first model *S*_vb_ = *S*_u_.

In the first model, auxin is assumed to reside either in the collective of transport channels in each of the vascular bundles (called a transport tube), or in the remaining tissue surrounding these tubes. The gross anatomical representation is shown in Fig. 3A. The model describes the change in time of the longitudinal distribution *u*(*x*,*t*) of tritium-labelled auxin (mol m^–1^) in all transport tubes taken together, at position *x* measured from the donor end, and the longitudinal distribution *y*(*x*,*t*) in the remaining tissue. The dynamics of *u* are given by an advection–diffusion equation,

$$\frac{\partial u}{\partial t}=D_{\text{u}}\frac{\partial^{2}u\;}{\partial x^{2}}-V\frac{\partial u}{\partial x},\;\;\;\;\;\;\;\;\;\text{for}\;\;x\;\text{ in }\;\left(0,L\right).$$ (1)

The first term on the right-hand side models diffusion of auxin in the longitudinal direction within the collective of transport tubes (called the *u*-compartment) with effective diffusion constant *D*_u_. The second term—the advection term—is responsible for the active unidirectional polar flow of auxin, with effective velocity *V. L* denotes the length of the stem segment. Auxin transport in the surrounding tissue (called the *y*-compartment) is modelled by diffusion only:

$$\frac{\partial y}{\partial t}=D_{\text{y}}\frac{\partial^{2}y}{\partial x^{2}},\text{ for }x\text{ in }\left(0,L\right).$$ (2)

Note that there is no exchange of auxin between the transport tube and surrounding tissue in this first model. The total net influx *J*_du_ (mol s^–1^) of auxin into all transport tubes at the donor end is given by

$$J_{\text{du}}=P_{\text{du}}^{+}S_{\text{u}}C_{\text{d}}-P_{\text{du}}^{-}u\left(0\right).$$ (3)

The first term represents influx from the donor well in which the labelled auxin is present at concentration *C*_d_. *S*_u_ is the area of contact of all transport tubes with the donor well and *P* ^*+*^_du_ represents the effective permeability (m s^–1^) of the combined system of cell membrane, cell wall, and possibly cell debris resulting from cutting the segment that is in between the buffer medium in the donor well and the first active auxin-transporting cells in the transport channels. The second term models reflux into the donor well with effective permeability *P* ^*–*^_du_. A similar expression is used for the net influx *J*_dy_ into the *y*-compartment, now with permeabilities *P* ^*+*^_dy_ and *P* ^*–*^_dy_. *S*_y_=*S–S*_u_ is the remaining part of the cross-sectional area of the stem segment.

Because of the experimental protocol, in which the receiver well is regularly emptied and replaced by plain buffer, the reflux of auxin from the receiver well back into the stem segment is negligible. Hence the net efflux *J*_ru_ of auxin from the *u*-compartment into the receiver well and similarly *J*_ry_ from the *y*-compartment, is modelled as

$$J_{\text{ru}}=P_{\text{ru}}^{-}u\left(L\right),\;J_{\text{ry}}=P_{\text{ry}}^{-}y\left(L\right).$$ (4)

These net fluxes provide mathematically necessary flux boundary conditions at *x=*0 (donor end) and *x=L* (receiver end) to Equations (1) and (2) (see the Supplementary data at *JXB* online). The concentration in the donor well changes in time according to

$$V_{\text{d}}\frac{dC_{\text{d}}}{dt}=\;-J_{\text{du}}-J_{\text{dy}},$$ (5)

Here *V*_d_ denotes the volume of the donor well. The accumulated amount of labelled auxin that has reached the receiver well up to time *t*, *N*_r_(*t*), is determined by

$$\frac{dN_{\text{r}}}{dt}=J_{\text{ru}}+J_{\text{ry}},$$ (6)

Figure 3B gives a schematic presentation of this model. The efflux profiles of standard PAT experiments could be fitted well with a small transport cost function (see Fig. 3C). The tissue profiles after 600min were a clear misfit (Fig. 3D), since the model predicted substantially smaller amounts in the subsegments than observed.

Biologically, two reasons for this accumulation can be given: first, auxin may become immobilized inside the transport tubes, after which it slowly remobilizes. Secondly, auxin may leak from transport tubes to surrounding tissue within the vascular bundles, where it diffuses, eventually possibly returning to the transport tube, for example facilitated by auxin influx carriers of the AUX1/LAX family.

To investigate the first hypothesis, a third variable *z*(*x*,*t*) was added to the model that describes the longitudinal density (mol m^–1^) of immobilized auxin. Immobilization of auxin occurs at a rate κ_1_ and remobilization at a rate κ_2_. Equation (2) for *y* is retained, now complemented by

$$\frac{\partial u}{\partial t}=D_{\text{u}}\frac{\partial^{2}u}{\partial x^{2}}-V\frac{\partial u}{\partial x}-\kappa_{1}u+\;\kappa_{2}z,$$ (7)

$$\frac{\partial z}{\partial t}=\;\kappa_{1}u-\;\kappa_{2}z.$$ (8)

**Fig. 4.**
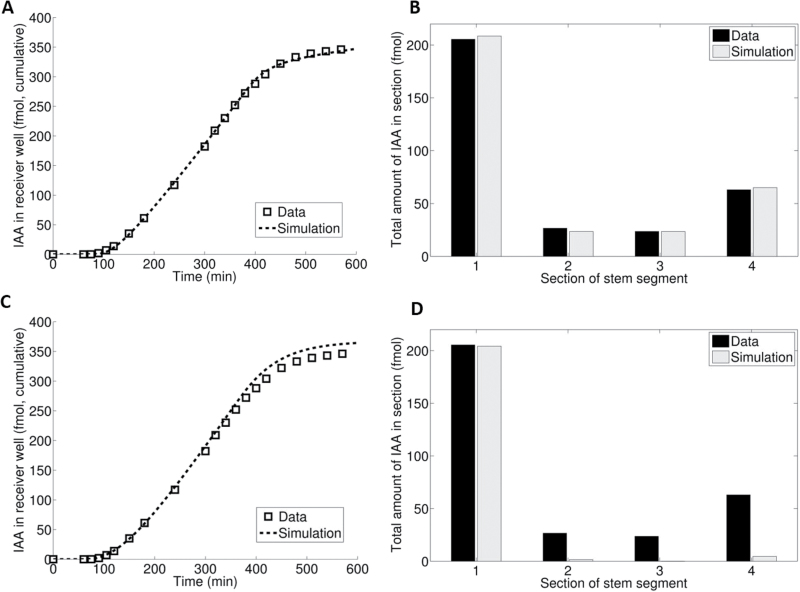
Fit of extended models to efflux and tissue profiles. (A) Fit of the (*u*,*y*,*z*) model (with immobilization) to the extended PAT efflux profile and (B) to the tissue profile after 600min. (C) Fit of the (*u*,*w*,*y*) model (with leakage to tissue surrounding PAT cells) to the extended PAT efflux profile and (B) to the tissue profile after 600min.

This model yielded an optimal fit to both efflux profile for extended PAT (see the Materials and methods) and tissue profile after 600min; see Fig. 4A and B.

For the second hypothesis, the initial model was extended with a third variable *w*(*x*,*t*) that models the longitudinal density of auxin outside the transport tubes, but within the vascular bundles. So *y* describes auxin density in the non-vascular tissue of stem segment in this case. Auxin diffuses in this *w*-compartment. At a rate *a*, auxin moves from the *u*- to the *w*-compartment. It returns at a rate *b*. The new equations for *u* and *w* are

$$\frac{\partial u}{\partial t}=D_{\text{u}}\frac{\partial^{2}u}{\partial x^{2}}-V\frac{\partial u}{\partial x}-au+\;bw,$$ (9)

$$\frac{\partial w}{\partial t}=D_{\text{w}}\frac{\partial^{2}w}{\partial x^{2}}+au-bw.$$ (10)

The *w*-compartment is also in contact with the donor and receiver well. The net influx into the *w*-compartment at the donor and efflux at the receiver is modelled similarly to Equations (3) and (4) as

$$J_{\text{dw}}=P_{\text{dw}}^{+}S_{\text{w}}C_{\text{d}}-P_{\text{dw}}^{-}w\left(0\right),\;J_{\text{rw}}=P_{\text{rw}}^{-}w\left(L\right).$$ (11)

Here *S*_w_=*S*_vb_–*S*_u_ is the area of the vascular bundles (*S*_vb_) that is not involved in the active transport of auxin. Additional terms *J*_dw_ and *J*_rw_ are added to the right-hand side of Equations (5) and (6).

This model also provided a fit to the efflux profile of extended PAT with small transport cost function (Fig. 4C). However, it poorly predicted the tissue profiles at 600min after extended PAT, as it computed an almost complete emptying of the stem segment (Fig. 4D). Apparently, the second modification should be rejected, while the first (*u*,*z*,*y*) model seems fully supported by the data.

However, we subjected the Arabidopsis *aux1/lax* quadruple loss-of-function mutant for all four AUX1/LAX1–LAX3 influx carriers to extended PAT experiments. This mutant has an almost wild-type phenotype (Bainbridge *et al*., 2008). The resulting transport profiles were quite different from those of the wild type, but the *u*,*z*,*y*-model still provided an optimal fit (see the Supplementary data at *JXB* online). Compared with the parameter settings for the wild type, mainly the parameter κ_1_ (i.e. the immobilization rate within the *u*-compartment) had to be changed by an almost 7-fold increase (see the Suplementary data). A biologically reasonable explanation for this increase in view of the function of the AUX1/LAX influx carriers could not be given however. Therefore, the *u*,*z*,*y*-model was also dismissed.

### Ultimate model

The ultimate model in this study is a model that combines both immobilization in transport tubes and leakage to surrounding tissue within the vascular bundle. It consists of the following system of partial and ordinary differential equations:

$$\frac{\partial u}{\partial t}=D_{\text{u}}\frac{\partial^{2}u}{\partial x^{2}}-V\frac{\partial u}{\partial x}-au+\;bw-\;\kappa_{1}u+\kappa_{2}z,$$ (12)

$$\frac{\partial w}{\partial t}=D_{\text{w}}\frac{\partial^{2}w}{\partial x^{2}}+au-bw,$$ (13)

$$\frac{\partial y}{\partial t}=D_{\text{y}}\frac{\partial^{2}y}{\partial x^{2}},$$ (14)

$$\frac{\partial z}{\partial t}=\;\kappa_{1}u-\;\kappa_{2}z.$$ (15)

$$V_{\text{d}}\frac{dC_{\text{d}}}{dt}=\;-J_{\text{du}}-J_{\text{dw}}-J_{\text{dy}},$$ (16)

$$\frac{dN_{\text{r}}}{dt}=J_{\text{ru}}+J_{\text{rw}}+J_{\text{ry}}.$$ (17)

Auxin influxes and effluxes for *u*, *w*, and *y* at the donor and receiver end remain unchanged. We assume *P* ^*−*^_rw_ *=P* ^*−*^_dw_ and *P* ^*−*^_ry_ *=P* ^*−*^_dy_. The partial differential equations (12)– (14) are complemented with boundary conditions

$$D_{\text{u}}\frac{\partial u}{\partial x}\left(0\right)-Vu\left(0\right)=\;-J_{\text{du}},\;\;\;\;\;\;\;\;\;D_{\text{u}}\frac{\partial u}{\partial x}\left(L\right)-Vu\left(L\right)=\;-J_{\text{ru}}$$ (18)

$$D_{\text{w}}\frac{\partial w}{\partial x}\left(0\right)=\;-J_{\text{dw}},\;\;\;\;\;\;\;D_{\text{w}}\frac{\partial w}{\partial x}\left(L\right)=\;-J_{\text{rw}},$$ (19)

$$D_{\text{y}}\frac{\partial y}{\partial x}\left(0\right)=\;-J_{\text{dy}},\;\;\;\;\;\;\;\;D_{\text{y}}\frac{\partial y}{\partial x}\left(L\right)=-J_{\text{ry}}.$$ (20)

### Simulation of data sets obtained from the extended PAT assay

**Fig. 5.**
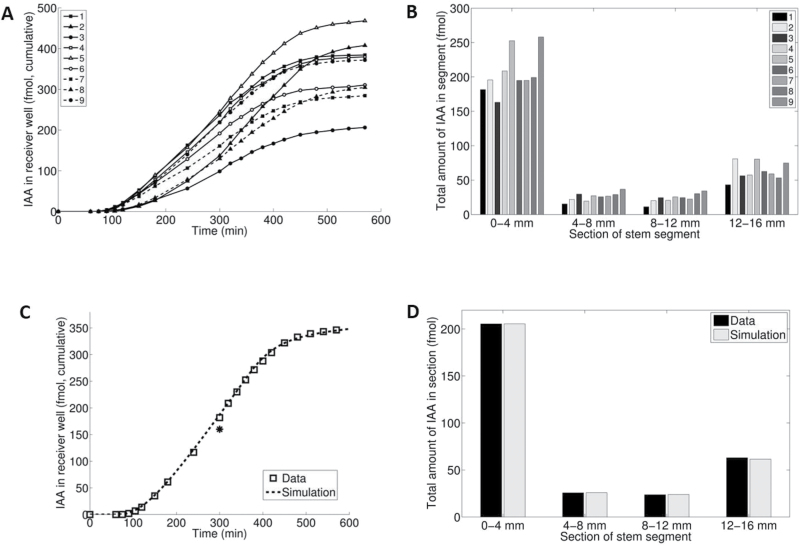
Efflux profiles from extended PAT. (A) Efflux profiles of nine individual wild-type stem segments and (B) their corresponding tissue profiles. (C) Extended efflux profile of the pooled data from the above nine individual stem segments and (D) the corresponding pooled tissue profile simulated using the ultimate model. An asterisk denotes the moment when the donor was replaced by plain MA medium.

Simulations of experimental data by means of the ultimate model were performed using the numerical COMSOL Multiphysics software program. The model was tested for its ability to fit quite a number of experiments optimally. As an example, we will discuss the data set from a representative experiment, using the extended PAT assay, which allowed measurement of individual transport profiles. The set of efflux profiles shown in Fig. 5 immediately gives an impression of the quantitative variation between individual stem segments. Such a variation was always observed, whatever attempts were made to standardize culture methods and experimental procedures.

We were able to simulate such sets of individual transport profiles by varying parameters as is shown in Table 1. The parameters are arranged as follows: (i) parameters governing the longitudinal densities *u*(*x*,*t*), *w*(*x*,*t*), *y*(*x*,*t*), and *z*(*x*,*t*), namely *D*_u_, *D*_w_, *D*_y_, *V*, *a*, *b*, κ_1_, and κ_2_ [see Equations (12)–(14)]; (ii) parameters governing the boundary-flux conditions at the donor end, namely *P* ^+^_du_, *P* ^+^_dw_, *P* ^+^_dy_, *P* ^–^_du_, *P* ^–^_dw_, and *P* ^–^_dy_, and at the receiver end: *P* ^–^_ru_, *P* ^–^_rw_ and *P* ^–^_ry_ [see Equations (3), (4), and (11)]; and (iii) anatomical parameters, namely: *S*, cross-sectional area of a stem segment; *S*_vb_, sum of cross-sectional area of vascular bundles; and α, cross-sectional area of the *u*-compartment expressed as fraction of *S*_vb_.

**Table 1.** *Parameter values used to simulate the experimental data sets of nine individual wild-type stem segments (1–9) and the pooled data (see* Fig. 4) Average values and the SD for the nine individual simulations are also shown. The cost function measures the quality of fit (see the Materials and methods, and the Supplentary data at *JXB* online).

Parameters	1	2	3	4	5	6	7	8	9	Average	SD	Pooled	Sensitivity
*D*_u_ (×10^–10^ m^2^s^–1^)	2.0	1.9	1.7	1.9	1.7	1.8	1.8	1.2	2.0	1.78	0.24	2.0	–12.4
*D*_w_ (×10^–10^ m^2^s^–1^)	0.7	0.7	0.7	0.7	0.7	0.7	0.7	0.7	0.7	0.70	0	0.7	–0.28
*D*_y_ (×10^–10^ m^2^s^–1^)	0.5	0.5	0.5	0.1	0.2	0.2	0.5	0.2	0.2	0.32	0.17	0.4	
*V* (×10^–6^ m s^–1^)	5.0	3.0	3.4	4.6	4.2	4.8	4.5	3.0	4.2	4.08	0.76	4.1	20.9
*a* (×10^–4^ s^–1^)	2.0	2.0	2.0	2.0	2.0	2.0	2.0	2.0	2.0	2.0	0	2.0	–6.93
*b* (×10^–4^ s^–1^)	7.0	7.0	7.0	7.0	7.0	7.0	7.0	7.0	7.0	7.0	0	7.0	8.6
κ_1_ (×10^–5^ s^–1^)	3.7	3.0	8.0	6.1	6.0	8.0	8.3	8.1	7.7	6.54	2.0	7.5	–4.94
κ_2_ (×10^–5^ s^–1^)	0.3	0.5	0.1	1.0	1.0	0.1	0.7	2.2	0.3	0.69	0.66	1.4	–1.45
*P* ^+^_du_ (×10^–6^ m s^–1^)	4.5	3.5	3.2	4.7	5.0	4.2	3.3	4.2	4.3	4.1	0.63	4.1	0.725
*P* ^+^_dw_(×10^–6^ m s^–1^)	4.5	3.5	3.2	4.7	5.0	4.2	3.3	4.2	4.4	4.1	0.64	4.0	–4.03
*P* ^+^_dy_(×10^–6^ m s^–1^)	0.5	0.4	0.5	0.8	0.6	0.5	0.5	0.5	0.6	0.5	0.13	0.5	
*P* ^–^_du_ (×10^–8^ m s^–1^)	1.5	1.5	1.5	1.5	1.5	1.5	1.5	1.5	1.5	1.5	0	1.5	0.005
*P* ^–^_dw_ (×10^–8^ m s^–1^)	1.5	1.5	1.5	1.5	1.5	1.5	1.5	1.5	1.5	1.5	0	1.5	0.784
*P* ^–^_dy_ (×10^–8^ m s^–1^)	1.5	1.5	1.5	1.5	1.5	1.5	1.5	1.5	1.5	1.5	0	1.5	
*P* ^–^_ru_ (×10^–8^ m s^–1^)	1.9	1.7	2.4	2.4	2.0	2.4	2.2	3.4	3.2	2.4	0.57	2.9	9.96
*P* ^–^_rw_ (×10^–8^ m s^–1^)	1.5	1.5	1.5	1.5	1.5	1.5	1.5	1.5	1.5	1.5	0	1.5	
*P* ^–^_ry_ (×10^–8^ m s^–1^)	1.5	1.5	1.5	1.5	1.5	1.5	1.5	1.5	1.5	1.5	0	1.5	
α=*S*_u_/*S*_vb_ %	33	32	30	33	33	33	30	31	35	32.2	1.6	32	1.77
*S* (×10^–7^ m^2^)	3.4	4.6	3.0	3.3	4.2	3.7	3.3	3.6	4.4	3.72	0.55	3.72	
*S*_vb_ (×10^–7^ m^2^)	0.6	0.9	0.6	0.6	0.7	0.6	0.7	0.6	0.7	0.69	0.1	0.69	
Value cost function γ	4.0	8.0	2.0	3.6	7.4	2.4	2.1	2.4	4.7			2.3	

The sensitivity is the dimensionless quantity given by the relative change in the cost function relative to the relative change in the parameter, or the partial derivative of the cost function in the direction of the parameter divided by the ratio γ/*p*, where *p* is the parameter.

*C*_d_=1×10^–7^ M. For further details, see text.

As a routine, *S* and *S*_vb_ were directly measured in each experiment (see the Materials and methods). A rough estimate of α was made from the cross-sectional area of the marked cells in the vascular bundles as revealed by the *AUX1-YFP* reporter (see Fig. 2). Fine-tuning of α was required in each simulation. From the definition of the *u*-, *w*-, and *y*-compartments given in the Supplementary data at *JXB* online, it follows that: *S*_u_=α*S*_vb_; *S*_w_=*S*_vb_ *–S*_u_, and *S*_y_=*S*– (*S*_u_+*S*_w_); these derived parameters *S*_u_, *S*_w_, and *S*_y_ were used in the numerical simulations. For the other parameter values, as far as possible, we used data from the literature (see the Supplementary data).

Table 1 shows that the parameter values obtained from the simulation of the pooled data are quite close to the average parameter values obtained from the simulation of individual transport profiles. Hence, for a comparison of data sets we may safely reduce the data to the pooled data set as is shown in Fig. 5C and D. Of course, for a more detailed statistical analysis, we need the parameter values from individual stem segments.

Before discussing the parameters in further detail at the end of this section, we will first comment upon the additional compartments *w* and *z.*

### The *w*-compartment: tissue surrounding the transport channels

The *w*-compartment in the model accounts for the exchange of auxin between the transport tubes and the surrounding tissue. From a mathematical point of view, this compartment could in principle be inside the *u*-compartment too. The quadruple mutant allows us to draw the conclusion that the *w*-compartment lies outside, as we shall now explain.

**Fig. 6.**
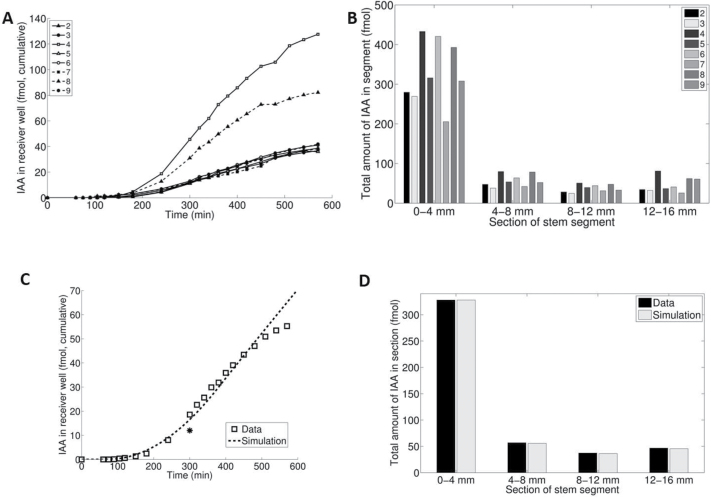
Efflux profiles from the extended PAT. (A) Efflux profiles of eight individual *aux1/lax1-3* quadruple mutant stem segments and (B) their corresponding tissue profiles. (C) Extended efflux profile of the pooled data from the above eight individual stem segments and (D) the corresponding pooled tissue profile simulated using the ultimate model. An asterisk denotes the moment when the donor was replaced by plain MA medium.

The data set and fitted parameter values for the quadruple mutant (Fig. 6; Table 2) can be directly compared with data and fit for the wild type (Fig. 5; Table 1). Characteristic deviations for the quadruple mutant as compared with the wild-type transport profile were: (i) the transport capacity is much less (see efflux profiles); (ii) after removal of [^3^H]IAA from the donor well at 300min, the efflux profiles show less bending between 300min and 600min; and (iii) the tissue profiles lack the relatively strong accumulation of auxin at the receiver end.

**Table 2.** *Parameter values used to simulate the experimental data sets of nine individual* aux1/lax1-3 *quadruple-mutant stem segments (2–9) and the pooled data (see* Fig. 5) Average values and the SD for the nine individual simulations are also shown.

Parameters	2	3	4	5	6	7	8	9	Average	SD	Pooled	Sensitivity
*D*_u_ (×10^–10^ m^2^ s^–1^)	1.2	1.2	1.3	1.0	1.1	0.9	1.0	1.8	1.19	0.28	1.2	–2.99
*D*_w_ (×10^–10^ m^2^ s^–1^)	0.7	0.7	0.7	0.7	0.7	0.7	0.7	0.7	0.70	0	0.7	–3.14
*D*_y_ (×10^–10^ m^2^ s^–1^)	0.6	0.2	0.9	0.1	0.2	0.2	0.8	0.5	0.44	0.31	0.47	
*V* (×10^–6^ m s^–1^)	3.4	3.6	4.2	3.1	4.5	5.5	4.3	3.8	4.05	0.75	4.0	13.2
*a* (×10^–4^ s^–1^)	2.0	2.1	2.0	2.2	2.5	2.0	2.6	2.2	2.2	0.24	2.2	–9.26
*b* (×10^–4^ s^–1^)	0.25	0.22	0.34	0.23	0.26	0.27	0.41	0.27	0.28	0.06	0.26	0.61
κ_1_ (×10^–5^ s^–1^)	9.0	7.5	6.0	8.0	20	30	10	8.0	12.3	8.4	10	–5.1
κ_2_ (×10^–5^ s^–1^)	1.0	1.0	1.0	1.0	0.5	0.5	1.0	0.5	0.81	0.26	1.0	0.51
*P* ^+^_du_ (×10^–6^ m s^–1^)	0.1	0.1	0.1	0.1	0.1	0.1	0.1	0.1	0.1	0	0.1	–0.04
*P* ^+^_dw_ (×10^–6^ m s^–1^)	4.6	3.2	4.2	4.0	4.2	4.7	4.3	3.9	4.14	0.47	4.2	–4.95
*P* ^+^_dy_ (×10^–6^ m s^–1^)	0.2	0.1	0.2	0.1	0.3	0.1	0.3	0.2	0.188	0.1	0.13	0.0004
*P* ^–^_du_ (×10^–8^ m s^–1^)	1.5	1.5	1.5	1.5	1.5	1.5	1.5	1.5	1.5	0	1.5	1.38
*P* ^–^_dw_ (×10^–8^ m s^–1^)	1.5	1.5	1.5	1.5	1.5	1.5	1.5	1.5	1.5	0	1.5	
*P* ^–^_dy_ (×10^–8^ m s^–1^)	1.5	1.5	1.5	1.5	1.5	1.5	1.5	1.5	1.5	0	1.5	2.87
*P* ^–^_ru_ (×10^–8^ m s^–1^)	2.0	2.5	2.5	2.9	2.9	3.5	2.4	1.5	2.53	0.61	2.9	
*P* ^–^_rw_ (×10^–8^ m s^–1^)	1.5	1.5	1.5	1.5	1.5	1.5	1.5	1.5	1.5	0	1.5	
*P* ^–^_ry_ (×10^–8^ m s^–1^)	1.5	1.5	1.5	1.5	1.5	1.5	1.5	1.5	1.5	0	1.5	
α=*S*_u_/*S*_vb_ %	29	32	30	28	26	32	27	30	29.3	2.2	28	1.92
*S* (×10^–7^ m^2^)	4.3	6.1	7.0	4.5	5.1	3.7	5.4	5.2	5.15	1.0	5.15	
S_vb_ (×10^–7^ m^2^)	0.9	1.1	1.5	1.1	1.1	0.7	1.0	1.1	1.06	0.23	1.1	
Value cost function γ	5.0	1.0	20	6.6	6.0	5.0	20	2.5			5.0	

*C*_d_=1×10^–7^ M. For the definition of sensitivity seethe footnotes of Table 1. For further details, see text.

The parameter values from the simulation of transport profiles are shown in Table 2. Apart from a few other differences with respect to the wild type, interestingly the values for the parameters *P* ^+^_du_ and *b* had to be set much lower than those of the wild type. As we have explained previously, these parameters govern the influx of auxin from the donor well into the *u*-compartment and the lateral influx of auxin from the *w*- into the *u*-compartment, respectively. This is in agreement with the role that is generally ascribed to the AUX1/LAX proteins. Since these auxin influx carriers are localized on the PM, positioning the *w*-compartment outside the *u*-compartment appeared to be a logical solution. Interestingly, in this quadruple mutant, no effect on the velocity parameter *V* was observed.

### The *z*-compartment: immobilization of auxin in the transport channels

**Fig. 7.**
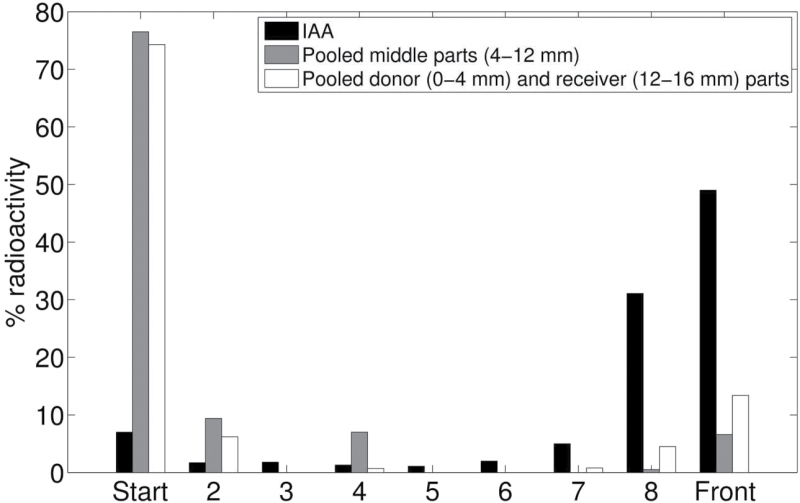
Thin-layer chromatography analysis. TLC for [^3^H]IAA, extracts of radioactive IAA from the middle parts, and pooled donor and receiver parts of stem segments at the end of an extended PAT after 600min. Most of the radioactivity (>85%) consisted of [^3^H]IAA derivatives with high retention values in both the pooled middle parts and the pooled donor and receiver ends.

The *z*-compartment was added to the model out of necessity to account for the non-negligible retention of auxin within the segments during extended PAT. In plants there are several enzymatic pathways that inactivate or immobilize auxin, for example by conjugation (Woodward and Bartel, 2005). In order to obtain at least a first impression of the fate of IAA during PAT, we extracted the radioactivity from the segments at the end of an extended PAT assay. Subsequent TLC revealed that by far most of the radioactivity (>85%) consisted of [^3^H]IAA derivatives with high retention values (Fig. 7). Although further identification of these derivatives and unravelling the pathway(s) by which they are formed is still a task for future research, it is quite clear that we may not neglect immobilization of auxin during its transport.

### The parameters and their interpretation

#### Velocity

A validation of methods to determine the velocity of PAT as they are described in the literature is given in Kramer *et al.* (2011). This validation rests on the definition of PAT given in Mitchison (1980). This is a clear choice, but we should realize that a definition of PAT is not as straightforward as it seems (see the Supplementary data at *JXB* online). Nevertheless, we will here follow Kramer *et al.* (2011) in validating our results. The methods described thus far include: the intercept method, in which the velocity is derived from the intersection of the steady-state part of the efflux profiles with the time axis; the measurement of the displacement of the edge of an auxin front at half maximum; the measurement of the first signal arriving at some fixed distance from an applied radioactive source; and the measurements of the displacement of the maximum of an auxin pulse. Kramer *et al.* (2011) consider the pulse method the most reliable of these methods. However, they regret the absence thus far in the literature of a method to derive the velocity from whole efflux profiles (i.e. including the transients) which of course should require some model to do that. Hence, for the first time we present here model-based estimates of PAT velocity.

Let us again take as an example the wild-type data set as we have presented it in Fig. 5 and Table 1. From the simulation of the pooled data, we obtained *V*=4.1×10^–6^ m s^–1^ (1.48cm h^–1^). Note that this value was obtained from the simulation of the whole data set, namely efflux plus tissue profiles. Next we tested *in silico* the above-mentioned methods to determine the velocity using the whole parameter set of the pooled data. Table 3 summarizes the results. *In silico*, the method of the first-arrived signal gives a value of *V* that corresponds nicely to the model-based estimate of 4.1×10^–6^ m s^–1^.

**Table 3.** Determination of the velocity of PAT using different methods

	Time of arrival	Length of segment	Velocity
Simulation transport profiles		1.6×10^–2^ m	4.1×10^–6^ ms^–1^
First signal simulation	4000 s	1.6×10^–2^ m	4.0×10^–6^ ms^–1^
First signal data	4500 s	1.6×10^–2^ m	3.56×10^–6^ ms^–1^
*In silico* pulse		1.6×10^–2^ m	3.94×10^–6^ ms^–1^
*In silico* front		1.6×10^–2^ m	Cannot be determined

**Fig. 8.**
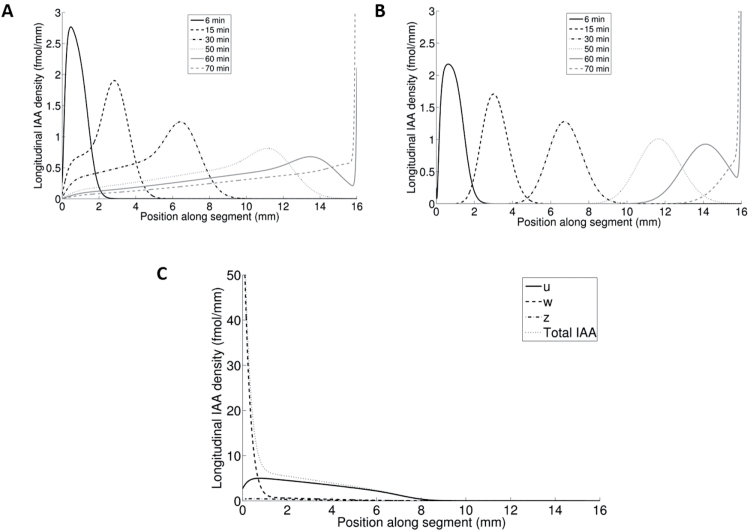

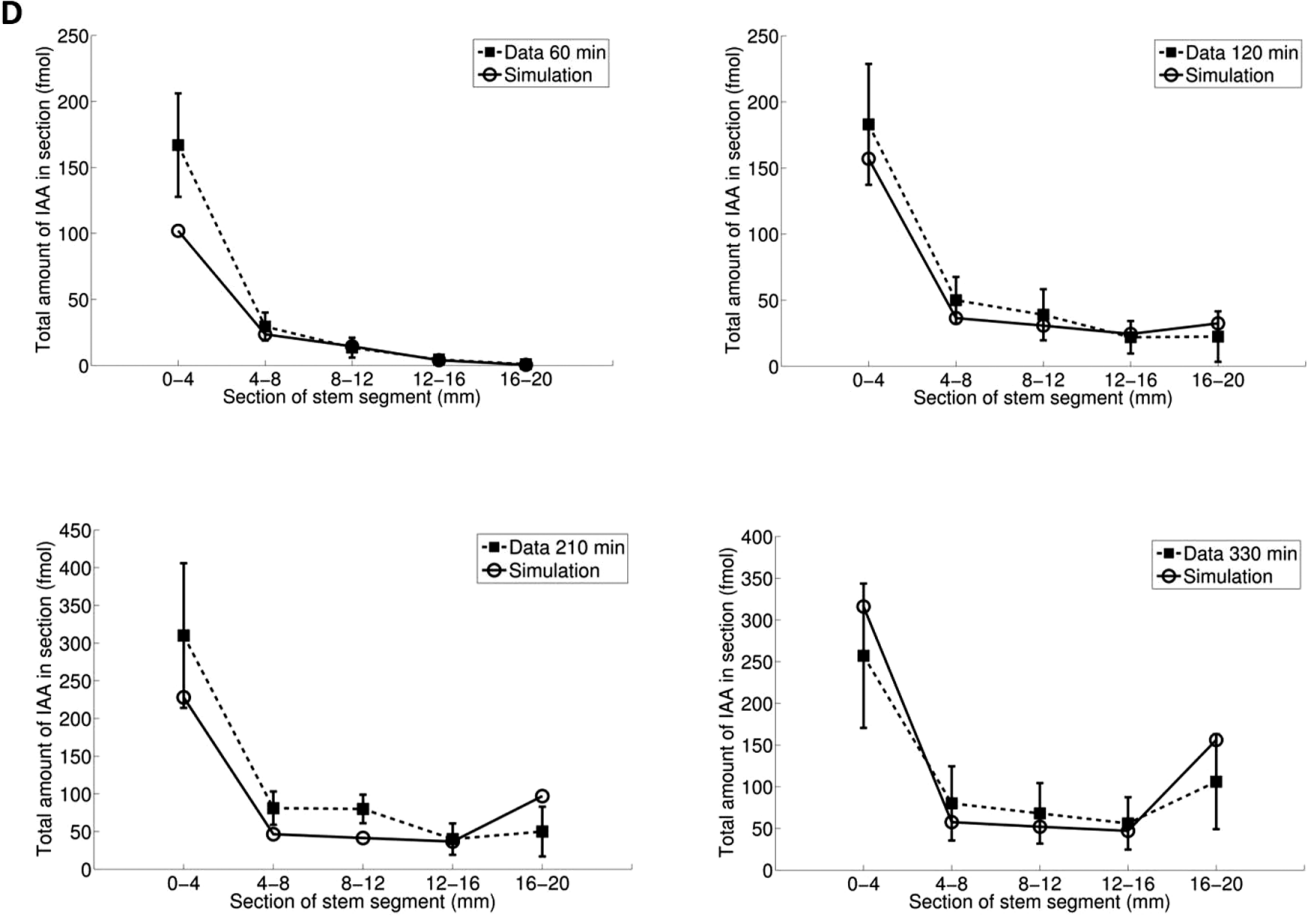
*In silico* experiments. (A) *In silico* 5min pulse experiment with the ultimate model for wild-type parameter settings in comparison with (B) the initial (*u*,*y*) model. Distribution of the total amount of auxin in the transport system in the stem segment (*u*+*w*+*z*) is shown at the indicated time points. (C) *In silico* experiment with the ultimate model (wild-type parameter setting): movement of an auxin front through a stem segment captured after 30min. No relevant maximum for estimation of the pulse propagation speed is available. (D) Comparison of tissue profile data with simulated data obtained with the complete model at the indicated time points.

Figure 8A and Supplementary Movie S1 at *JXB* online show the movement of an auxin pulse along the *x*-axis of the *u*-compartment in a computer simulation with the complete model. The segment was fed during 5min from a virtual donor well containing 10^–4^ mol m^–3^ auxin. After a brief period lasting ~600 s, which more or less coincided with the loading of the system, displacement of the maximum of the pulse was practically linear with time up to ~14.5mm (the total length of the segment was 16mm), giving a velocity *V*=3.94×10^–6^ m s^–1^ (Table 3). If we followed the maximum of the pulse of total auxin (*u*+*w*+*z*), we found almost the same value of *V* (data not shown). Note that the pulse showed a conspicuous backward tail. Obviously this is due to the exchange (*w*-compartment) and immobilization (*z*-compartment) of auxin during its transport, since if we strip these additional compartments from the model, we obtain a ‘classical’ pulse as is shown in Fig. 8B. The maximum of this pulse moved with *V*=4.12×10^–6^ m s^–1^. Practically the same value was obtained from the displacement of the front at half maximum in the case where the segments were continuously fed with auxin (data not shown), which agrees with the theory (see also Kramer *et al.*, 2011). In the case of the complete model, however, not only was the shape of a pulse different, but the shape of the front of auxin moving through a segment was also different (Fig. 8C). In this case it was not possible to determine the velocity from the displacement of the front at half maximum because the front was too flat.

The above explanation about pulses and moving auxin fronts is based on the results of *in silico* experiments, but do their predictions agree with the real data? Up till now, we did not study pulses, but we studied how tissue profiles evolved in continuous-feeding experiments, which is the common practice in our standard PAT. We used 20mm long segments, so that we were able to study the movement of auxin over a somewhat longer trajectory before distortion occurs at the receiver interface. At various times, samples of stem segments were harvested and processed to determine the amount of [^3^H]IAA in 4mm intervals along the entire length of the segments. The obtained average tissue profiles were compared with a simulation using parameter values from Table 1. Figure 8D indicates that the prediction from the complete model shows a fair agreement with the data. Therefore, we tentatively concluded that the above-explained virtual experiments were not far off the reality.

In summary, macroscopic auxin transport velocity is a concept that can be defined well only within the context of a mathematical model (see the Supplementary data at *JXB* online). The values for *V* we thus obtained over a significant series of experiments are within the range of 1–1.5cm h^–1^, which are regularly found in the literature (see Kramer *et al.*, 2011, AuxV database). The velocity with which simulated auxin pulses move through segments corresponds nicely to the *V* we obtained with our model. However, it is not practical to perform these pulse experiments to determine *V*. Measurement of the arrival time of the first signal also appears to give a fair estimate of velocity. In practice, however, this method is quite unreliable because of sampling intervals and the threshold formed by the background value (signal to noise ratio). In addition, exact solutions to the advection–diffusion equation have a concentration greater than zero for all positions and at all times after *t*=0. Thus, there is no useful time of first arrival in the exact case.

#### Exchange between the transport channels and the surrounding tissue

The exchange between the *u*- and *w*-compartments is governed by the phenomenological parameters *a* and *b* (dimension s^–1^). The necessity for this exchange was clearly demonstrated during the analysis of the *aux1/lax* quadruple mutant. We developed a model for the interpretation of these parameters, based upon assumptions on the processes for auxin transport between cells in the *u*-compartment, the intermediate apoplast, and cells in the neighbouring *w*-compartment (see the Supplementary data at *JXB* online). Under the assumption that the *u*-compartment consists of the xylem-associated parenchyma, we obtain

$$a=\;\frac{\Gamma }{S_{\text{u}}}\cdot \frac{P_{\text{u}}^{-}P_{\text{w}}^{+}}{P_{\text{u}}^{+}+P_{\text{w}}^{+}},\;\;\;\;\;\;\;\;\;\;\;b=c\cdot \frac{\Gamma }{S_{\text{u}}}\cdot \frac{P_{\text{u}}^{+}P_{\text{w}}^{-}}{P_{\text{u}}^{+}+P_{\text{w}}^{+}},$$ (21)

where *c=S*_u_/*S*_w_, Γ denotes the total circumference of the *u*-compartment (in xylem parenchyma) in a cross-section, *P* ^+^_u_ is the effective permeability of the cell membrane of a cell in the *u*-compartment for auxin from the apoplast into that cell, and *P* ^–^_u_ is the permeability in the opposite direction. *P* ^+^_w_ and *P* ^–^_w_ are defined similarly for the *w*-compartment (i.e. the total of xylem vessels). These represent the permeabilities in either direction of the xylem vessel wall.

From cross-sections, we estimated that the ratio of the contribution to the total area of vascular bundle made by xylem (*S*_xl_) compared with phloem (*S*_ph_) is 57–43%. We refitted the pooled transport curves for the wild type and quadruple mutant, by fixing *S*_u_ to the values found before (see Tables 1 and 2), while modifying *S*_w_ such that *S*_u_+*S*_w_ equals *S*_xl_=0.57×*S*_vb_=4.0×10^–8^ m^2^ as obtained from cross-sections for the wild type. This results in *c*_wt_=1.11 and *S*_y_=*S*–*S*_xl_=3.3×10^–7^ m^2^. It turned out that an optimal fit could be obtained by fixing all other parameters as in Table 1 (pooled data), except for putting *P* ^*+*^_dw_=1.1×10^–5^ m s^–1^ and *P* ^*+*^_dy_= 4.45×10^–7^ m s^–1^. For the *aux1/lax* quadruple mutant we assumed the same contribution of xylem and phloem to the vascular bundles. A similar approach led to *S*_xl_=6.3×10^–8^ m^2^, *c*_qd_=1.11, and *S*_y_=4.5×10^–7^ m^2^ for the *aux1/lax* quadruple mutant. Also in this case all other parameters could be fixed as in Table 2, except for putting *P* ^*+*^_dw_=1.1×10^–5^ m s^–1^ and *P* ^*+*^_dy_= 1.3×10^–7^ m s^–1^.

Assume further that Γ/*S*_u_, *P* ^*–*^_u_, *P* ^*+*^_w_, and *P* ^*–*^_w_ are the same for the wild type and the quadruple mutant; that is, neither the structure of the PAT channels within the xylem, nor the transport of auxin out of PAT cells, nor the permeability of xylem vessel walls is influenced by the *aux1/lax* loss of function. Then we obtain from Equation (21), that

$$\frac{b_{\text{qd}}}{b_{\text{wt}}}=\;\frac{a_{\text{qd}}}{a_{\text{wt}}}\cdot \frac{c_{\text{qd}}}{c_{\text{wt}}}\cdot \frac{P_{\text{u},\text{qd}}^{+}}{P_{\text{u},\text{wt}}^{+}}.$$ (22)

Substituting the fitted values for *a* and *b* from Table 1 and 2 yields

$$\frac{P_{\text{u},\text{qd}}^{+}}{P_{\text{u},\text{wt}}^{+}}=\frac{0.3\times 10^{-4}s^{-1}}{7\times 10^{-4}s^{-1}}\cdot \frac{2\times 10^{-4}s^{-1}}{2.2\times 10^{-4}s^{-1}}\cdot \frac{1.11}{1.11}\approx 0.04.$$ (23)

Thus, the AUX/LAX auxin import carriers implement a 25-fold increase in membrane permeability.

#### Accumulation at the donor and the receiver boundary

The parameters that govern the uptake and accumulation at the donor boundary are *P* ^+^_dy_, *P* ^+^_du_, and *P* ^+^_dw_. While *P* ^+^_dy_ accounts mainly for the accumulation in the non-transporting tissue at the donor boundary, the more interesting values are those of *P* ^+^_du_ and *P* ^+^_dw_, which are directly involved in the loading of the transport channels. The relatively high value for *P* ^+^_du_ (4.1×10^–6^ m s^–1^) corresponds well to the results of Rutschow *et al.* (2014), who found a value of 1.5×10^−6^ m s^–1^ for the influx of auxin mediated by AUX1 using protoplasts from Arabidopsis roots. In the quadruple mutant, *P* ^+^_du_ dropped ~41-fold to a value of 1×10^–7^ m s^–1^ (see Table 2). In the wild type, *P* ^+^_dw_ has about the same value as *P* ^+^_du_. However, it was not affected in the quadruple mutant (compare Tables 1 and 2). At this stage, we simply do not understand such a high value of *P* ^+^_dw_, in particular not for a *w*-compartment that is expected to reside in the xylem parenchyma of the vascular bundles. Does it result from adsorption of the sticky auxin molecules at the lignified apoplast, or is it a modelling artefact? Anyway, the model as it stands requires such a high value of *P* ^+^_dw_ for loading of the PAT system. A satisfactory explanation for its high value and its significance awaits further anatomical clarification of the *w*-compartment.

Supplementary Movie S2 at *JXB* online shows a time sequence of a simulated advancing auxin front in the *u*-compartment using the parameters from Table 1. This gives a strong impression of what happens at the boundary between segment and receiver well. There, the front bumps against the transport barrier at that interface, whereupon there is a relatively rapid accumulation of auxin in the last few tenths of a millimetre (see for example Fig. 8A).

Two important parameters that influence the accumulation at the receiver boundary are *P* ^–^_ru_ and *D*_u_. We could compensate for either an increase or a decrease of *D*_u_ by a corresponding increase or decrease of *P* ^–^_ru_. Hence, within the range of either a 3.5-fold increase or decrease of *D*_u_, its effect on the accumulation of auxin at the receiver boundary could be compensated for by an equal increase or decrease of *P* ^–^_ru_, showing that within that range the ratio of *D*_u_ over *P* ^–^_ru_ is the major determining factor.

**Fig. 9.**
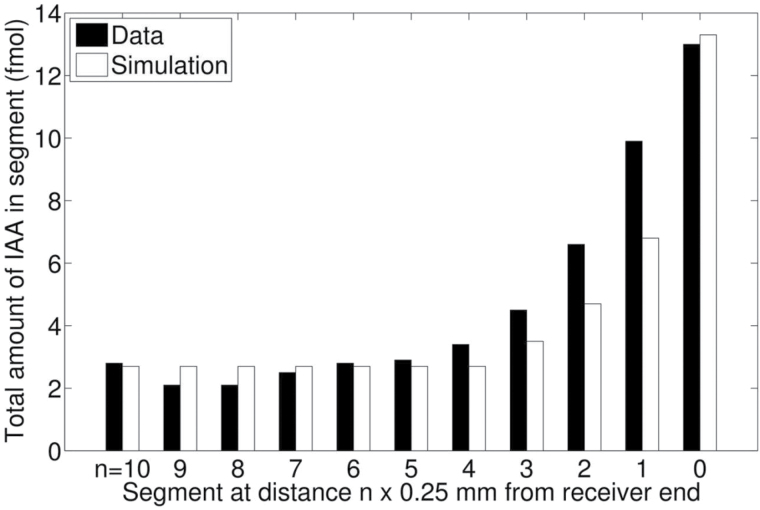
Accumulation at the receiver boundary. Tissue profile after a standard PAT where the last 3mm of the receiver ends of stems were cut into 250 μm long sections. Data were simulated by a linear increase of parameter *a* over the last 1.5mm of the stem segments with a factor 16.

Interestingly, a mathematical analysis of the steady-state auxin flux in the MGGM model through a cell file, in which the flux is reduced in the last transport barrier in the last cell, shows that accumulation occurs only in about the last two cells (computation not shown). In order to verify this prediction, we decided to study the accumulation of auxin in the last 4mm by cutting 1mm sections. We found that in fact the accumulation occurred in the last 1.5mm (xylem-associated parenchyma cells have a typical measured length of ~80–100 μm). This was further confirmed by a type of experiment in which, after an incubation period of 300min, fresh last 4mm pieces were cut into a complete series of 250 μm thick sections. Each section in the series was immediately processed for counting radioactivity. Interestingly, the data not only showed that the accumulation occurred in the last 1.5mm, but also showed a positive gradient towards the segment and receiver boundary (Fig. 9). We could not explain such a type of gradient by the current model, not even by increasing *D*_u_ up to 7×10^–10^ m^2^ s^–1^ and a corresponding increase of *P* ^–^_ru_.

A first attempt to approximate the observed tissue profile required an extension of the model with in particular a positive gradient of the parameter *a* towards the segment and receiver interface over the last 1.5mm. Figure 9 shows a simulation of the observed tissue profile with the extended model. As we have explained before, the parameter *a* governs the flux of auxin from the *u*- into the *w*-compartment. The interesting question now arises of whether the observed gradient in auxin accumulation at the receiver boundary is brought about by increasing peripheral instead of predominant basal orientation towards the wounded surface of PIN proteins in PAT cells. Such an reorientation of PIN proteins at wounded surfaces has been described in the literature (Sauer *et al.*, 2006) and we have observed it ourselves for PIN3–GFP proteins above basal wounded surfaces in inflorescence stem segments of Arabidopsis (data not shown).

#### Immobilization of auxin in the transport channel

**Fig. 10.**
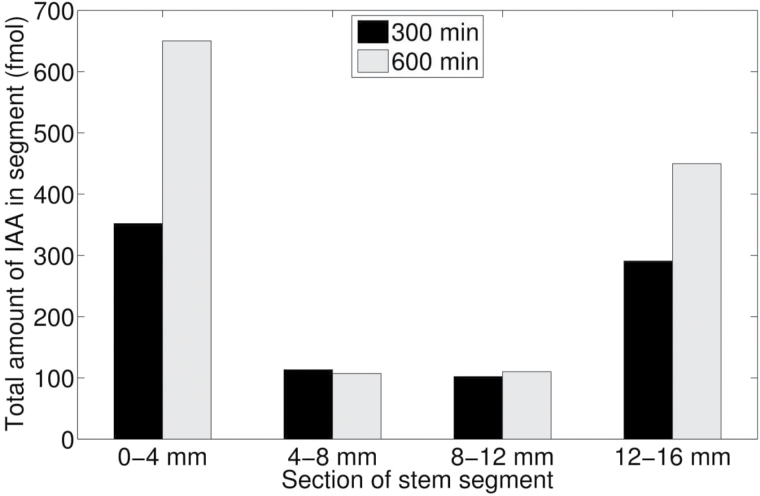
Comparison of 300min and 600min standard PAT tissue profiles. Tissue profiles after a standard PAT taken after either 300min or 600min.

The parameters κ_1_ and κ_2_ served quite well in accounting for interpreting the observed tissue profiles in the extended PAT. However, the *z*-compartment is a simplified representation of possible pathways leading to immobilization, for example by conjugation (Woodward and Bartel, 2005). Figure 10 shows two tissue profiles from standard PAT, one obtained after an incubation period of 300min and the other one after an incubation period of 600min. We found no increase of *z* in the middle parts (4–12cm) of the stem segments during 300min and 600min, but *z* continued to increase at the donor and receiver boundaries. Our model does not yet account for this observation. As we will explain in the next section, we had to take care of the continuing immobilization at the receiver boundary for proper estimates of the steady-state flux of auxin through the PAT system.

#### Concentration dependence of PAT

**Fig. 11.**
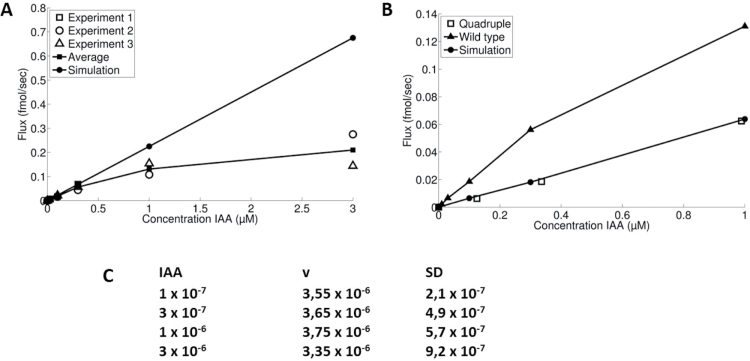
Concentration dependence of PAT. (A) Concentration dependence of the steady-state fluxes in wild-type and (B) in quadruple mutant stem segments. (C) Effect of increasing IAA donor concentrations on the velocity (*v*) of PAT for wild-type.

The model is linear; that is, no possible saturation kinetics are included. However, the possible involvement of auxin influx and efflux carriers suggests that at least some parts of the PAT system may be saturable. The question then of course is: which parts? In order to try to answer this question, we performed a number of experiments covering donor concentrations ranging from 1×10^–8^ M to 3×10^–6^ M. The results from these experiments are summarized in Fig. 11. It shows the influence of different donor concentrations on the steady-state fluxes through the PAT system. These fluxes were estimated as follows: for each donor concentration, tissue and efflux profiles of the pooled data from the nine segments in each batch in the standard PAT (incubation time 300min) were simulated by the model. We let the computer calculate: (i) the flux of auxin through the segment by multiplying the velocity and the concentration of auxin (mol m^–1^) in the *u*-compartment (after 300min in the 4–12mm part of the segments); and (ii) the flux of auxin into the receiver well between 300min and 600min, and accumulation of auxin between 300min and 600min at the receiver boundary. Subsequently we let the computer calculate the steady-state flux by adding these two quantities and dividing by 300min.

Assuming conservation of auxin we would expect that the two calculations should agree.

As an example let us take the parameter values from the pooled data shown in Table 1. The donor concentration was 10^–4^ mol m^–3^ (1×10^–7^ M). The amount of auxin released into the receiver well between 300min and 570min was 310.5fmol. During this interval, the accumulation of radioactive auxin increased with 54.5fmol at the receiver boundary. Hence, the total flux of [^3^H]IAA through the PAT system during the 270min interval was 310.5+54.5=365fmol, giving a steady-state flux of 365/270×60=0.0225fmol s^–1^.

The simulation revealed that the steady-state concentration of auxin in the *u*-compartment between the donor and receiver ends of the segments (4–12mm) was 5.75×10^–12^ mol m^–1^. The velocity was 4.1×10^–6^ m s^–1^. This gives a steady-state flux F_steady_=*v*×*ū*=0.0236fmol s^–1^, which is consistent with the previous estimate.

As expected, the model predicts that without changing parameter values, the steady-state fluxes are directly proportional to the donor concentrations (Fig. 11A). However, simulation of the real data from experiments with different donor concentrations required a systematic lowering of the values of *P* ^+^_du_ and *b* with donor concentrations >3×10^–7^ M. This resulted in a substantial relative reduction of the steady-state fluxes as is shown in Fig. 11A (line with filled squares), suggesting that some part of the transport system became saturated. Since we had to lower the values for *P* ^+^_du_ and *b* systematically, we assumed that possible auxin import carriers were responsible for the saturation. In order to test this possibility, we performed an experiment with the auxin influx carrier *aux1/lax1-3* quadruple mutant. Figure 11B shows that within the donor concentrations tested (ranging from 1×10^–7^ M to 1×10^–6^ M), we found that, in contrast to the wild type, the steady-state fluxes followed the predicted straight line, suggesting that the auxin influx carriers of the AUX1/LAX1–LAX3 family are responsible for the saturation of the PAT system.

Interestingly, we did not find an effect of the donor concentrations on the velocity except for some random variation between samples (Fig. 11C).

## Discussion

As outlined in the Introduction, our direct aim was to develop an experimental system that satisfies the following requirements: (i) the system must allow direct measurements of PAT and must be derived from model plants with a variety of accessible putative PAT mutants, such as Arabidopsis; and (ii) There must be an appropriate mathematical model for the system to interpret and evaluate the obtained real data.

We have achieved the following.

(i) We developed a simple efficient donor–receiver assay based on not too short stem segments (16mm) from inflorescences of the model plant Arabidopsis, providing a reproducible and well controlled experimental environment for measuring characteristic features of PAT.
(ii) A single advection–diffusion equation as model for the transport of auxin through a stem segment, as suggested by the MGGM model, cannot describe the characteristic features of transients in standard PAT and our extended PAT assays. Instead, we extended the model by adding immobilization and exchange of auxin with surrounding tissue during PAT, where each step in the extension of the advection–diffusion model was validated by experimental results. The model that was finally obtained (the ultimate model) has an optimal fit to the transients in PAT and extended PAT assays. It allows accurate estimation of transport velocity and steady-state fluxes through the transport system.
An optimal fit was achieved using a Gradient Descent method, where the step size is selected by loose linear search. The landscape of the cost function, which measures the quality of fit, turned out to be quite complicated as a function of the parameters. It had some quite local minima in which the algorithm could get stuck and provide a suboptimal solution. By combining manual fit for obtaining a good initial ‘guess’ of the parameter setting with this automated algorithm we could determine a parameter setting that results in a fit that is within the experimental accuracy, being locally optimal. In view of the local minima in the cost function landscape, it is questionable whether a more advanced optimization algorithm (Newton or quasi-Newton method) with an arbitrary starting point would perform better. This also obstructs investigation of the full parameter space in search of a globally optimal fit. Moreover, it seems to require the improvement or development of new statistical and numerical techniques for parameter estimation in time-dependent systems of coupled partial and ordinary differential equations by specialists in this field, since recent work covers apparently time-independent situations only (see, for example, Bui-Thanh and Girolami, 2014; De los Reyes, 2015). This is clearly beyond the scope of this study.
(iii) The ultimate model provides an adequate theoretical platform for interpreting experimental transport data, also from putative transport mutants. As an example, we gave a comprehensive account of the *aux1/lax* quadruple mutant, in which all four genes encoding PAT-associated import carriers were knocked out. Only assuming exchange of auxin with the tissue surrounding PAT cells gave a satisfactory explanation of the different PAT characteristics of the quadruple mutant, thereby confirming a function for the import carriers in preventing leakage of auxin from the PAT channels.
(iv) Since the ultimate model enables accurate estimation of velocity and steady-state fluxes through the system, we were able to study dose–response relationships satisfactorily using the standard PAT assay. We found that the steady-state flux of auxin through the PAT system exhibits saturation kinetics whereby at up to 3×10^–7^ M IAA, the fluxes are approximately linearly dependent on the donor concentration. Saturation kinetics could be simulated in the model by manipulating the exchange parameters, in particular by a progressive decrease of the import parameter *b*, whereas over the entire range of donor concentrations up to 3×10^–6^ M IAA the velocity *v* was not altered. These findings suggest that possibly the import carriers are responsible for the observed saturation kinetics. This is in good agreement with published data that show that AUX1 saturates at a comparable concentration of ~0.8×10^–6^ M IAA (Yang *et al.*, 2006). This is sustained by the observation that, in contrast to the wild type, the steady-state flux of auxin in the quadruple mutant is linearly dependent on the donor concentration up to 1×10^–6^ M, while the velocity remained unaltered, as in the wild type.

Our ultimate model describes PAT at the macroscopic level—the stem segment. Nevertheless, it models particular structures within: an active transport compartment; an enveloping compartment into which auxin leaks and in which it can diffuse; and a domain in which auxin is immobilized. It does not assume any specific mechanisms for cell–cell transport that are responsible for the active transport, leakage, or immobilization. In principle, different mechanisms at a lower level may result in the same system of equations describing PAT at the macroscopic level. However, the mathematical expression of phenomenological parameters such as *a*, *b*, or *V* in the latter model in terms of parameters in models for the underlying mechanisms will be different for different assumptions. A change in lower level parameters, caused, for example, by mutation, will yield different changes in macroscopic parameters. This allows testing of hypothetical lower level mechanisms through careful analysis of macroscopic data in view of the model and the expression for parameters that link the micro with the macro level.

Nevertheless there are major issues that still need to be resolved. The distribution of auxin and the mechanisms of transport within PAT cells and of cell–cell transport in PAT channels are still poorly known. We have pointed out that there is no direct experimental evidence for the specific assumptions made by the MGGM model, in spite of the fact that they seem reasonable and plausible. Moreover, the MGGM model is a simplification at the level of cell tissue, which ignores both exchange of auxin with the tissue surrounding PAT cells and immobilization of auxin. Our results show that these processes cannot be neglected when interpreting all important aspects of PAT data. First, in describing the shape of transients in transport curves and, secondly, when interpreting the movement of an auxin pulse through the stem segment, as our simulations suggest. The additional processes result in a strong deformation of the shape of the pulse during transport, changing a nicely bell-shaped curve into a long-tailed stretched ‘bump’ on the way.

In the more recent literature, mechanisms other than simple diffusion have been proposed for intracellular transport of auxin in PAT cells. Our finding with PAT in internodal cells of *Chara corallina* (Boot *et al.*, 2012) with relatively high velocities (4–5cm h^–1^) argues against simple diffusion, at least in these giant cells with a length of at least 3–5cm. An obvious candidate for an alternative to simple diffusion would be transport in vesicles via the actin cytoskeleton or cytoplasmic streaming (Friml and Palme, 2002; Baluska *et al.*, 2003). Thus far, however, both in *Chara* and in higher plants including Arabidopsis, accepted specific inhibitors of cytoplasmic streaming were without effect. Recently, transport vesicles were found in Arabidopsis which were not described previously and which are rapidly transported in a myosin-XI-dependent manner (Peremyslov *et al.*, 2013). These vesicles could be candidates for intracellular distribution and transport of auxin.

In the MGGM model, it is assumed that transport of auxin from cell to cell in a PAT channel is via the intermediate apoplastic interfaces. These form open couplings between the cells: auxin can leak by radial diffusion from these interfaces. If, indeed, the AUX1/LAX1–LAX3 transporters serve in also keeping the steady-state concentration of auxin in the apoplastic interface between PAT cells as low as possible in order to prevent substantial leakage, then in view of the MGGM model we may be surprised that in the *aux1/lax* quadruple mutant we did not find an effect on the effective velocity. We may wonder why nature has not selected closed connections, for example plasmodesmata (White *et al.*, 2011).

Our present research is focused on the following issues: first, screening of a substantial number of PAT mutants of Arabidopsis and interpreting the data in view of the current macroscopic model. Over the past few years, a number of genes have been characterized whose protein products are believed to play an important role in PAT. Plants that have mutations in these genes have been tested for the effects on PAT using a variety of different bioassays. None of these bioassays, however, satisfies the full requirements for direct PAT measurements as formulated in our present study. The outcomes of our screening will be compared with data and conclusions from the literature (a follow-up paper is in progress). The screening may reveal points at which the current model needs refinement or adjustment; for example, the accumulation and immobilization of auxin at the receiver boundary.

Secondly, the mathematics defining the present macroscopic model will be developed further, including a mathematically sound derivation of correspondences between macroscopic, mesoscopic, and microscopic parameters. Alternative mechanisms for intra- and intercellular transport in PAT channels will thus be investigated by combining mathematics and experiment (a first paper is in progress).

## Supplementary data

Supplementary data are available at *JXB* online.

The Supplementary data show a detailed account of the development of the macroscopic mathematical model for polar auxin transport in inflorescence stem segments of *Arabidopsis thaliana* that is presented in the main text. The ultimate model resulted from an experiment-driven step-by-step extension of a single advection–diffusion equation for auxin transport as suggested by the MGGM model based on the chemiosmotic theory. We also explain the method of parameter optimization that was used.

Movie S1. Movement of an auxin pulse (5min) along the *x*-axis of the *u*-compartment in a computer simulation using parameters from Table 1.

Movie S2. Time sequence of a simulated advancing auxin front in the *u*-compartment using the parameters from Table 1.

Supplementary Data
