# Supplementary material for: Modelling the dynamics of polar auxin transport in inflorescence stems of Arabidopsis thaliana
Source: J Exp Bot. 2015 Nov 2;67(3):649–66. doi: 10.1093/jxb/erv471 (PMC4737066; doi:10.1093/jxb/erv471)
Supplement: Supplementary Data [file supp_67_3_649__index.html]

Modelling the dynamics of polar auxin transport in inflorescence stems of Arabidopsis thaliana — Modelling the dynamics of polar auxin transport in inflorescence stems of Arabidopsis thaliana — Modelling the dynamics of polar auxin transport in inflorescence stems of Arabidopsis thaliana — Supplementary Data 

# Modelling the dynamics of polar auxin transport in inflorescence stems of *Arabidopsis thaliana*

## Supplementary Data

Data files

- Supplementary\_data\_Development\_of\_mathematical\_modelling.pdf - Supplementary Data
- Movie\_1.avi - Supplementary Data
- Movie\_2.avi - Supplementary Data
