# Supplementary material for: Modelling the dynamics of polar auxin transport in inflorescence stems of Arabidopsis thaliana
Source: J Exp Bot. 2015 Nov 2;67(3):649–66. doi: 10.1093/jxb/erv471 (PMC4737066; doi:10.1093/jxb/erv471)
Supplement: Supplementary Data [file supp_erv471_Supplementary_data_Development_of_mathematical_modelling.pdf]

Supplementary material to: ‘Modelling the dynamics of polar auxin transport in inflorescence stems of *Arabidopsis thaliana*’

Kees J.M. Boot, Sander C. Hille, Kees R. Libbenga,
Lambertus A. Peletier, Paulina C. van Spronsen,
Bert van Duijn and Remko Offringa

Contents

1 The concept of auxin transport velocity **3**

2 Parameter values in the literature **5**
- 2.1 Diffusion constants 5
- 2.2 Membrane permeabilities 6
- 2.3 Observed transport velocities 6

3 A family of macroscopic models for PAT **7**
- 3.1 General modeling assumptions 7
- 3.2 Model I: a single transport tube 9
- 3.3 Model IIa and IIb 12
  - 3.3.1 Model IIa: Immobilization 12
  - 3.3.2 Model IIb: Exchange of auxin with surrounding tissue 15
- 3.4 Model III: Combined immobilization and exchange 17

4 Microscopic interpretation of exchange rates **18**

5 Parameter optimization **23**
- 5.1 Cost function and good fit 23
- 5.2 Parameter optimization algorithm 24
- 5.3 Implementation 26
- 5.4 Discussion 27

In this Supplementary Material (SM) we present a detailed account of the development of the ultimate macroscopic mathematical model for polar auxin transport in inflorescence stem segments of *Arabidopsis thaliana* that is presented in the Main Text. In fact, the ultimate model resulted from an experiment-driven step-by-step extension of a single advection-diffusion equation for auxin transport as suggested by the mathematical model developed by Mitchison [23], Goldsmith, Goldsmith and Martin [12], simply called the MGGM model, based on the chemiosmotic theory for auxin transport [28, 25, 27].

We start with discussing issues that arrise when defining a concept of auxin transport velocity at the macroscopic level of a stem segment (see Section 1). These issues have a general character. They are not bound to a specific model.

Our objective for modelling PAT is beyond simply describing the data comprehensively. We also aim at gaining insight into the microscopic mechanisms at cellular level that are responsible for polar transport of auxin. An experimentally validated model at macroscopic level together with availability of suitable transport mutant plants and well-designed experiments with these allow limiting the possible mechanisms at microscopic level or estimating microscopic parameter values. In Section 4 we shall provide an initial result of this approach. The necessary 'bridging of scales' from microscopic mechanisms and parameters to effective macroscopic equations and parameters is mathematically challenging. It is beyond the scope of this paper to discuss this in detail. Nevertheless, some particular derivations of this sort will appear in this Supplementary Material.

Section 2 discusses values of relevant physiological parameters as found in the literature. These are a starting point for simulations, fitting and development of the ultimate macroscopic model for PAT. Section 3 documents the development of these continuum models and the modeling decisions made in more detail than found in the Main Text. These are based upon the ability of these models to fit the experimental data with parameter values or changes therein that are biologically interpretable. The model that ultimately resulted from this approach (Model III) is discussed in Section 3.4. Section 4 provides an example how some insights into microscopic mechanisms can be obtained from the interpretation of macroscopic data for wild-type and suitable mutant plants in view of mathematical models at macroscopic and microscopic scale. Section 5 concludes this Supplementary Material with a detailed account of the used approach to parameter optimization.

We choose to use SI units everywhere from the start by convention. In this way we avoid recomputing parameter values and simulation results to different units. At some points we shall quote values from the literature. We then state the value in the units as found in the reference together with the value in SI units when needed.

1 The concept of auxin transport velocity

Introducing the concept of auxin transport velocity at the spatial scale of a stem segment (i.e. the macroscopic scale) is not as straightforward as it might seem, see also [20]. Mitchison [23] considers the velocity of auxin transport as the velocity of position of the maximum value of a pulse of auxin within the stem segment. This concept cannot be applied to our experimental set-up of a transport experiment. We observe a moving front within the stem segment, which is getting less sharp while it is progressing through the segment, due to the smoothing effect of diffusive processes (see Figure 7, Main Text and SM, Movie 2). One may introduce transport velocity as the speed of the mid-level point in this widening front. However, 'mid-level' itself seems ill-defined.

An alternative is furnished by the speed at which the centre of mass of the auxin distribution within the stem moves. It is used by Mitchison [23] since it coincides with his definition for transport velocity applied to a pulse. However, for a moving front it will yield a value that is approximately a factor 2 lower than the intuitive velocity of the front.

The length of the segment divided by the time of first appearance of auxin at the other end may seem yet another alternative. However, the combined effects of diffusion and the experimental resolution at which auxin can be detected influence the time at which the auxin level in the receiver well is observed as being above background measurement noise. This makes this concept ill-determined. So this seems inappropriate too [20].

The issue is that for diffusion processes velocity is not well-defined, although *flux* is, i.e. the amount of auxin that is transported through the stem segment per unit of time. It is also (total) flux of auxin that is measured in the transport experiments. Using the cell-to-cell fluxes $\phi_{i,i+1}$, Mitchison introduced an alternative (cf. [23], equation (19), p.504), which we call the

average mass flow velocity:

$$V_{AMF} := \frac{\ell \sum_i \phi_{i,i+1}}{I}, \tag{1}$$

where I is the total auxin content of the system, ℓ is the length of a single transport cell and $\phi_{i,i+1}$ is the flux from cell i to $i = 1$. V_{AMF} is the velocity of a homogeneous steady mass flow through the segment of a solution containing auxin at average density I/L such that the average flux through the system results:

$$V_{AMF} \cdot \frac{I}{L} = \frac{1}{N} \sum_i \phi_{i,i+1}. \tag{2}$$

Here N is the total number of cells. Thus, V_{AMF} results from comparison of the complex anatomy and transport processes within the stem segment effectively with transport by a steady fluid flow through a simple tube with no internal structure.

A neater approach towards defining transport velocity, as proposed by Kramer *et al.* in [20] (Supplementary Material), is to take also the effect of diffusion into account: the transport velocity is the velocity V that is obtained by fitting the advection-diffusion equation

$$\partial_t u = D\partial_x^2 u - V\partial_x u, \qquad \text{on } (0, L) \tag{3}$$

(with appropriate boundary conditions at $x = 0$ and $x = L$) to experimental data. V captures phenomenologically the net effect of all directed auxin transport processes, while the diffusion term combines all undirected processes.

In our work we improve on the proposition made by Kramer *et al.* by starting from a more complicated model than (3) for transport through the segment, because the experimental data we obtained could not be fitted to (3). See Section 3 for a detailed account of the development of our model together with the experimental motivation.

Thus, unlike auxin flux through the system, macroscopic auxin transport velocity is a concept that can be defined well only within the context of a mathematical model that simplifies the biophysical reality. In the case of V_{AMF} it is a transport equation for the equivalent 'fluid flow':

$$\partial_t u = -V_{AMF}\, \partial_x u. \tag{4}$$

Kramer *et al.* propose (3). We shall consider a more elaborate system of equations to determine velocity (namely equations (23)-(26)), among others forced by experimental data.

Equations (3), (4) or our model (23)-(26) provide an effective description of transport at macroscopic level, i.e. the stem segment, ignoring cellular structure by using a continuum approximation. Our model takes some aspects of the detailed anatomy of the stem segment into account though. It identifies different 'compartments' that correspond to parts of the vascular bundles. In that sense our model is intermediate between equations (3) and (4) and a description of auxin transport at cellular level, e.g. through a linear array of auxin-transporting cells, as furnished by chemiosmotic theory and the MGGM model. This theory has been developed in a series of papers [28, 25, 12, 18, 23]. Mitchison [23] provided a neat mathemematical model of the chemiosmotic theory and elaborate analysis of its properties.

2 Parameter values in the literature

In this section we discuss model parameters for which values could be established with confidence from the literature or from experiments that were conducted specifically to determine their values. We distinguish between those that are fixed by anatomy (e.g. cell geometry, tissue structure, etc.), physiology (e.g. permeabilities of membranes, acidity of particular parts of the cellular tissue, diffusion constants) and the experimental design. These parameter values are summarized in Table 1.

2.1 Diffusion constants

Mitchison [23] mentions a value of 6.7×10^{-6} cm^2/s $=$ 6.7×10^{-10} m^2/s for the diffusion constant for auxin in agar, quoting [21] on page 491. Kramer [15] mentions estimates derived from radio-label profiles of 0.04-0.09 cm^2/h, which equals $1.1 - 2.5 \times 10^{-9}$ m^2/s.

We performed diffusion experiments with tritium-labelled IAA in 2% agarose gels (data not shown). Calculation of the diffusion constant based on the radio-label profile after 1 and 5 hours resulted in 7×10^{-10} m^2/s. This is in good agreement with the value cited by Mitchison, so we take $D_u = 7 \times 10^{-10}$ m^2/s as our reference value.

Name:	Value:	Unit:	Description:
ℓ	1.0×10^{-4}	m	Average length of a transport cell
d_0	1.2×10^{-5}	m	Diameter of a transport cell
δ_m	5×10^{-9}	m	Thickness of cell membrane
δ_a	1×10^{-6}	m	Thickness of apoplast
d	7×10^{-10}	m^2/s	Diffusivity of IAA in cytoplasm
P_{AH}	5×10^{-7}	m/s	Permeability of cell membrane for IAAH through simple diffusion [7, 8, 17, 19]
pH_c	7.0	–	pH of cytoplasm [30, 23]
pH_a	5	–	pH of apoplast [22, 10, 23]
pK_a	4.8	–	Acidity constant of IAA [17, 23]
L	1.6×10^{-2}	m	Length of experimental stem segment
C_d	1.0×10^{-4}	mol/m^3	Total concentration of IAA in donor well
V_d	$1 - 2 \times 10^{-6}$	m^3	Volume of donor well

Table 1: *Orders of magnitude of anatomically, physiologically and experimentally fixed fundamental parameter values.*

2.2 Membrane permeabilities

Kramer [17], p.1234, reports a cell membrane permeability for protonated IAA of 0.2 cm/h $=$ 5.6×10^{-7} m/s. In [19] the authors refer to two uptake studies, one with mesophyll protoplasts from tobacco plants and the other with suspension-cultured tobacco cells that reveal a permeability of 0.14 cm/h $= 3.9 \times 10^{-7}$ m/s [7] and 0.18 cm/h $= 5.0 \times 10^{-7}$ m/s [8], respectively. For the anion form a value of 5×10^{-9} m/s was evaluated in this last study. They criticize values obtained in artificial membranes, which are one order of magnitude larger.

2.3 Observed transport velocities

See Kramer *et al.* [20] for an extensive discussion of the concept of auxin transport velicity and the AUX V database of measured velocities.

3 A family of macroscopic models for PAT

The chemiosmotic theory yields a mathematical model, the MGGM model, that consists of a linear array of discrete cells in which internally diffusion of IAA takes place and which are coupled through prescribed auxin flux between neighbouring cells in the array that depends on the auxin concentrations in these cells at either end [23]. Thus, it consists of a large system of partial differential equations (PDEs) of diffusion type, coupled by flux boundary conditions. There are as many equations as there are cells.

The purpose of this section is to arrive at a mathematical model for PAT in *Arabidopsis* in a minimal continuum formulation, i.e. in terms of a system of PDEs with the smallest number of equations as possible, minimally parametrized, that is able to describe well the measured IAA transport in wild type plants and some mutants. Actually, we shall describe a family of mathematical models of increasing complexity, where each step-wise increase in complexity is motivated by a misfit to experimental data in more detail than provided in the Main Text. Model III is the ultimate result of this study.

The simplest PDE model that we start with, is such that its solution approximates in space and time the solution to the mesoscopic model presented above. It turned out that it cannot explain some essential features of the experimental observations, although it gives a good fit to others. Our fitting procedure and algorithm is presented in Section 5.

As it turns out, in order to understand PAT in *Arabidopsis* one needs to incorporate more processes into the considerations than those included in the mesoscopic model based on chemiosmotic theory.

3.1 General modeling assumptions

For each of the models that we present we use the following general assumptions. The cross section of the *Arabidopis* stem segment at a distance x from its apical side, Ω_x, has fixed area S for all x. S_{vb} denotes the area of the vascular bundles within the cross section. Let $f_{vb} := S_{vb}/S$ be the fraction of the total cross section that consists of the vascular bundles. We assume that the PAT channels, i.e. the linear arrays of transport cells discussed in the mesoscopic model, are located within these vascular bundles. This assumption is motivated by the expression of PAT-markers in inflorescence

stems of *Arabidopsis* (see Figure 2, Main Text). The transport system is the total of all PAT channels through the stem segment. α represents the fraction of the cross section of the vascular bundles that corresponds to the directional auxin transport system.

Auxin, indole-3-acetic acid (IAA), is a weak acid with acidity constant $\mathrm{pK}_a = 4.8$ ($K_a = 1.58 \times 10^{-5}$ M; cf. [17]). Therefore it exists in both protonated form IAAH and anion form IAA^-, depending on proton concentration. We assume that the reactions $\mathrm{IAAH} \rightleftarrows \mathrm{IAA}^- + \mathrm{H}^+$ are fast compared to the other processes involved in PAT, such that we can assume quasi-steady state. The Henderson-Hasselbalch equation then allows to compute the fraction f of IAA in anion form at given pH or proton concentration h as

$$f = \frac{1}{1 + 10^{\mathrm{pK}_a - \mathrm{pH}}} = \frac{1}{1 + \frac{h}{K_a}} = \frac{K_a}{K_a + h}. \tag{5}$$

Table 2 provides the computed fractions of IAA in either form for some pH values.

pH	Fraction IAA^-	Fraction IAAH
4	0.137	0.863
5	0.613	0.387
7	0.994	0.006

Table 2: *Fraction of* IAA *in anion and protonated form for several* pH *values.*

The spatial distribution of all IAA at time t can then be described by a single local total concentration $C(x, \eta, \zeta; t)$ (mol/m^3). Here η and ζ denote spatial coordinates in a right-handed Cartesian coordinate frame that has its origin in the mid-point of the left-hand side of the stem segment, corresponding to the boundary with the donor well (see Main Text, Material & Methods). The positive x-axis is pointing through the middle of the segment to the boundary with the receiver well that is located at $x = L$. Since we shall be modeling in terms of longitudinal densities, the spatial variable η and ζ will be used temporarily only. Later they disappear in this exposition.

Note throughout the forthcoming discussion, that concentrations and longitudinal densities of auxin will refer to total auxin concentration or density, i.e. to the sum of both anion and protonated form.

3.2 Model I: a single transport tube

In this first model, we assume that all of the cells in a cross section of the vascular bundle that contain IAA are part of a PAT channel. Let Ω_x^u denote the corresponding subset of Ω_x and S_u its area. That is,

$$S_u := |\Omega_x^u| = \alpha \cdot f_{vb} \cdot S \tag{6}$$

for all $0 \leq x \leq L$.

Moreover, we assume that $C(x, \eta, \zeta; t)$ is approximately constant for $(\eta, \zeta) \in \Omega_x^u$. Then we can represent the total distribution of IAA by a longitudinal density function $u(x, t)$:

$$u(x,t) \approx \int_{\Omega_x^u} C(x, \eta, \zeta; t) d\eta d\zeta.$$

Accordingly, the part of the stem segment occupied by the auxin transport system (i.e. the collective of PAT channels) will be called 'u-compartment'.

We model the dynamics of u by means of an advection-diffusion equation, similar to [15, 20]:

$$\partial_t u = D_u \partial_x^2 u - V \partial_x u \qquad \text{on } (0, L), \tag{7}$$

where D_u and V represent the effective macroscopic diffusivity and transport velocity of IAA in the stem segment. Equation (7) is complemented with boundary conditions at $x = 0$ and $x = L$:

$$-[D_u \partial_x u(0) - V u(0)] \; = \; P_{du}^+ S_u C_d - P_{du}^- u(0) \; =: \; J_{du}, \tag{8}$$

$$-[D_u \partial_x u(L) - V u(L)] \; = \; P_{ru}^- u(L) \; =: \; J_{ru}. \tag{9}$$

Here C_d is the total concentration of IAA in the donor well, which is considered homogeneously distributed over the well. Because the receiver well is sampled regularly and then filled with fresh plain buffer medium, we ignore in (9) reflux of IAA from the receiver well into the receiver end of the stem segment.

At the donor end, not only the cells in the transport system are in contact with IAA in the donor well, but also the remaining cell tissue in the cross section. Let Ω_x^y be the part of the stem segment outside the transport system at position x. The uptake and distribution of IAA in this part of the stem segment is modeled with a longitudinal density $y(x, t)$:

$$y(x,t) \approx \int_{\Omega_x^y} C(x, \eta, \zeta; t) \, d\eta d\zeta.$$

We assume that y satisfies

$$\partial_t y = D_y \partial_x^2 y \tag{10}$$

and boundary conditions similar to (8)–(9):

$$-D_y \partial_x y(0) = P_{dy}^{+} S_y C_d - P_{dy}^{-} y(0) =: J_{dy}, \tag{11}$$

$$-D_y \partial_x y(L) = P_{dy}^{-} y(L) =: J_{ry}. \tag{12}$$

Here, $S_y = S - S_u$ is the area of Ω_x^y, which we assume to remain fixed, i.e. independent of x. Note that we state implicitly, that the processes that govern the efflux of IAA from the 'y-tissue' into the well are the same at donor and receiver end of the stem.

The concentration of IAA in the donor well changes according to

$$\frac{dC_d}{dt} = -\frac{1}{V_d}(J_{du} + J_{dy}). \tag{13}$$

Here V_d denotes the volume of the donor well. In practice radioactive IAA is super-abundant so that the decrease of radioactive IAA in the donor well can be neglected. However, in an extended PAT experiment (see Main Text, Material & Methods) the donor well is replaced by plain buffer medium after some time point. Some radioactive IAA in the stem segment then flows back into the donor well. With the dynamic modeling of C_d as in (13) it is possible to track this reflux.

The total amount of IAA, $N_r(t)$, that has reached the receiver well up to time t satisfies

$$\frac{dN_r}{dt} = J_{ru} + J_{ry}. \tag{14}$$

Figure 1 provides a schematic representation of Model I.

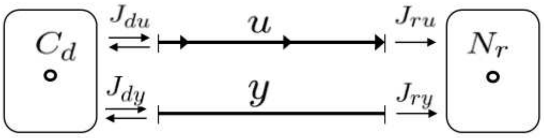

Figure 1: *Schematic representation of Model I. A solid line in the spatially distributed compartments u and y indicates diffusion. Arrows indicate the presence of an advection term and the direction of positive velocity.*

We take the following initial conditions:

$$u(x,0) = 0,\ y(x,0) = 0 \text{ for } 0 < x < L, \qquad N_r(0) = 0, \quad C_d(0) = C_{d0}.$$

Fitting results:
Table 3 shows the parameter values to fit Model I to the measured transport curve and tissue profile, from a representative experiment. See Figure 2 for the quality of fit to the transport profile. Measurement of the IAA content of the donor well yielded $C_{d0} = 0.107\ \mu\text{M} = 1.07 \times 10^{-7}\text{mol/m}^3$.

Fitted parameter:		Wild type
D_u	$(\times 10^{-10}\text{m}^2/\text{s})$	2
D_y	"	0.2
V	$(\times 10^{-6}\text{m/s})$	3.27
P_{du}^{+}	$(\times 10^{-6}\text{m/s})$	8.5
P_{dy}^{+}	"	0.232
P_{du}^{-}	$(\times 10^{-8}\text{m/s})$	1.5
P_{dy}^{-}	"	1.5
P_{ru}^{-}	$(\times 10^{-8}\text{m/s})$	2.2
P_{ry}^{-}	"	1.5
$\alpha = S_u/S_{vb}$	%	32.5
S	$(\times 10^{-7}\text{m}^2)$	7
S_{vb}	"	1.3

Table 3: *Fitted constants in Model I for nine wildtype stem segments. Fit based on standard PAT efflux profile and tissue profile at 600 min. S and S_{vb} were measured.*

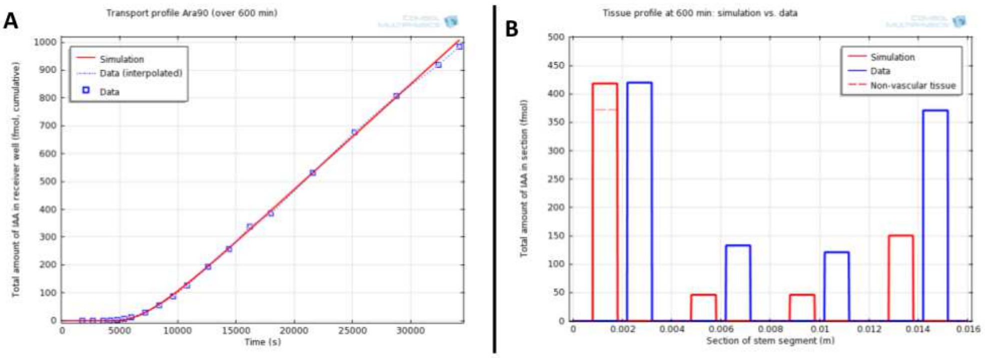

Figure 2: *Fit to Model I of experimental data of standard PAT efflux profile (A) and tissue profile (B) at 600 min of the average of nine stem segments. Corresponding parameter values are presented in Table 3. The lower part in the column representing the simulation result for the first 4 mm up to the dashed line indicates the amount of radioactive IAA found in the 'y-compartment', i.e. the tissue surrounding the transport system (the 'u-compartment').*

Discussion:
Figure 2A shows that, in general, one obtains excellent fits to measured efflux profiles, even over incubation periods of about 10 hrs. Figure 2B, however, reveals that the model underestimates the amount of IAA present in the middle part and the receiver end of the stem segment. This leads us to consider modifications to the model.

3.3 Model IIa and IIb

There are two extensions of Model I with the least number of additional variables and parameters, namely: adding immobilization of IAA or introducing leakage of IAA to neighbouring tissue combined with diffusion within that tissue. In order to discriminate better between these options we changed the standard PAT assay to an extended PAT assay (see Main Text, Materials & Methods). In addition, we employed a mutant line of *Arabidopsis* in order to reach a proper decision about which model provides the best fit. In this mutant, denoted Quadruple, the four genes *aux1/lax1-3* encoding for the AUX1/LAX influx carriers that reside all over the plasma membrane in transporting cells are knocked out.

3.3.1 Model IIa: Immobilization

Let $z(x,t)$ denote the longitudinal density of immobilized IAA within the transport system ('u-compartment') at position x at time t. The equations for u, z and y in this model are:

$$\partial_t u = D_u \partial_x^2 u - V\partial_x u - \kappa_1 u + \kappa_2 z, \tag{15}$$

$$\partial_t y = D_y \partial_x^2 y, \tag{16}$$

$$\partial_t z = \kappa_1 u - \kappa_2 z. \tag{17}$$

There is no diffusion in the z-equation (17) to represent immobilization. Note that immobilization is not permanent (if $\kappa_2 > 0$). Figure 3 shows a schematic representation of this model.

The boundary conditions for equations (15) and (16) are precisely (8) and (11) at 0 and (9) and (12) at L. The equations for the concentration C_d and $N_r(t)$ are the same as in Model I. The initial conditions for all dynamic variables are 0, except for $C_d(0) = C_{d0}$.

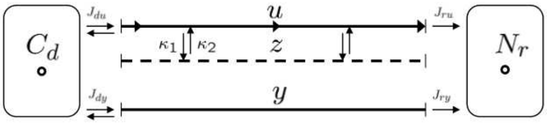

Figure 3: *Schematic representation of Model IIa. The dotted line indicates that z is immobile.*

Fitting results:
Table 4 presents the parameter setting used to obtain the fit of efflux and tissue profiles at the end of the efflux experiment as shown in Figure 4 for wild type and Figure 5 for the *aux1/lax* quadruple mutant.

Fitted parameter:		Wild type	Quadruple
D_u	$(\times 10^{-10}\text{m}^2/\text{s})$	2	2
D_y	"	0.2	0.7
V	$(\times 10^{-6}\text{m/s})$	3.0	2.7
κ_1	$(\times 10^{-5}/\text{s})$	6	40
κ_2	"	2	5
P^+_{du}	$(\times 10^{-6}\text{m/s})$	6.3	2.0
P^+_{dy}	"	0.52	0.49
P^-_{du}	$(\times 10^{-8}\text{m/s})$	1.5	1.5
P^-_{dy}	"	1.5	1.5
P^-_{ru}	$(\times 10^{-8}\text{m/s})$	2.7	8.0
$\alpha = S_u/S_{vb}$	%	61.5	62
Measured:			
S	$(\times 10^{-7}\text{m}^2)$	3.72	5.15
S_{vb}	%	0.69	1.07

Table 4: *Fitted parameters in Model IIa for the average over nine wild-type stem segments and eight quadruple aux1/lax1-3 mutant stem segments. Fit based on extended PAT efflux profile and tissue profile at 600 min* ($C_d(0) = 0.10\ \mu$M)*. S and S_{vb} are averages over the measured values.*

Discussion:
In general this model gave quite satisfactory fits to data from both standard and extended PAT assay, both for wild-type and quadruple mutant. The extended PAT reveals that quite a substantial amount of radioactivity was retained in the tissue. Extraction of the radioactivity from the segments at the end of the extended PAT and subsequent thin-layer chromatography revealed that by far (more than 85%) consisted of ^{3}H-IAA derivatives with high retention values (see Figure 6, Main Text).

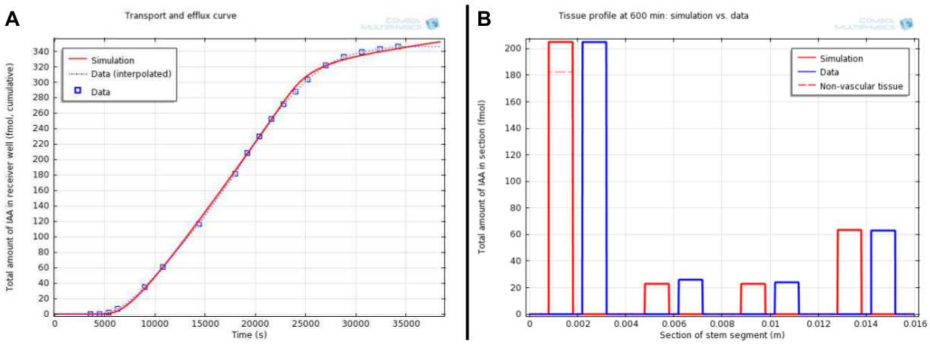

Figure 4: *Fit to Model IIa of experimental data for wild-type of extended PAT curve (A) and tissue profile (B) at 600 min (Average of nine stem segments of wild-type). Corresponding parameter values are presented in Table 4.*

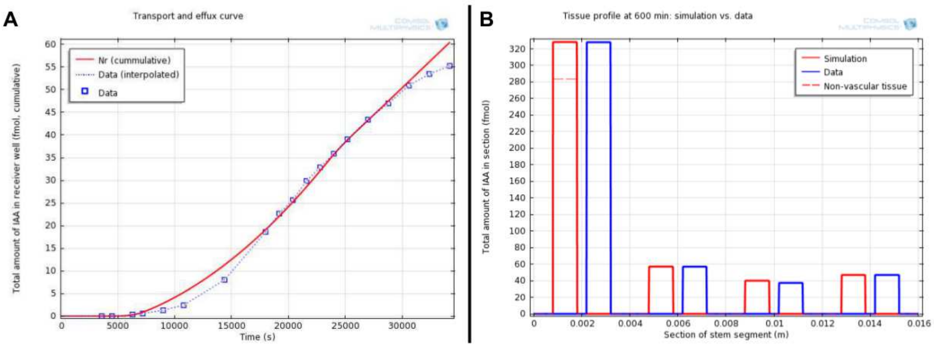

Figure 5: *Fit to Model IIa of experimental data for a quadruple mutant of efflux profile (A) and tissue profile (B) at 600 min (average of eight stem segments of quadruple aux1/lax1-3 mutant). Corresponding parameter values are presented in Table 4.*

It is the change in parameter value from wild-type to quadruple that is hard to interpret biologically. The most apparent change in parameter, i.e. the rise of κ_1 (see Table 4) – meaning an increase of the rate of immobilization of IAA, is hard to explain at molecular biological level. The quadruple mutant lacks AUX1/LAX1-LAX3 proteins. It is known that these are located on the plasma membrane and are involved in auxin *influx* [16].

Thus, although Model IIa can provide good fits to all experimental data, the biological interpretation of changes in parameter values between wild-type and quadruple mutant leads us to conclude that Model IIa is still not satisfactory.

3.3.2 Model IIb: Exchange of auxin with surrounding tissue

Instead of immobilization, we tried exchange of IAA between the PAT channel and surrounding tissue within the vascular bundle. In this tissue there is no effective active transport at macroscopic scale. In comparison with Model IIa, more parameters are needed to describe this case, since one has to provide a description for the uptake of IAA at donor end and release of IAA at receiver end.

We introduced a longitudinal density $w(x,t)$ for the total amount of IAA in the tissue surrounding the transport tube (called the 'w-compartment'). Within the stem segment the dynamics are given on $(0, L)$ by

$$\begin{aligned} \partial_t u &= D_u \partial_x^2 u - V \partial_x u - au + bw, && (18) \\ \partial_t w &= D_w \partial_x^2 w + au - bw, && (19) \\ \partial_t y &= D_y \partial_x^2 y, && (20) \end{aligned}$$

complemented by boundary conditions for u and y at 0 and L as in Model I and Model IIa, while for w we have

$$\begin{aligned} -D_w \partial_x w(0) &= P_{dw}^+ S_w C_d - P_{dw}^- w(0) \;=:\; J_{dw}, && (21) \\ -D_w \partial_x w(L) &= P_{dw}^- w(L) \;=:\; J_{rw}. && (22) \end{aligned}$$

Figure 6 shows the schematic representation of this model.

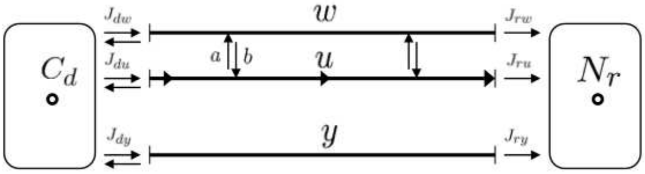

Figure 6: *Schematic representation of Model IIb.*

Fitting results:
Table 5 presents the parameter setting used to fit efflux and tissue profiles obtained from standard and extended PAT assays for the same experiment as used when fitting Model IIa (wild-type). The values for S and f_{vb} are the averages over the measured values for the nine samples in the series.

Discussion:
Figure 7B clearly shows that the tissue profile at the end of the extended PAT assay experiment at 600 min cannot be fitted, while the complete efflux

Fitted parameter:		Wild type
D_u	$(\times 10^{-10}\mathrm{m}^2/\mathrm{s})$	2
D_w	”	7
D_y	”	0.4
V	$(\times 10^{-6}\mathrm{m/s})$	4.2
a	$(\times 10^{-4}/\mathrm{s})$	2.0
b	”	7.0
P^+_{du}	$(\times 10^{-6}\mathrm{m/s})$	3.0
P^+_{dw}	”	3.0
P^+_{dy}	”	0.55
P^-_{du}	$(\times 10^{-8}\mathrm{m/s})$	1.5
P^-_{dw}	”	1.5
P^-_{dy}	”	1.5
P^-_{ru}	$(\times 10^{-8}\mathrm{m/s})$	2.0
P^-_{rw}	”	1.5
P^-_{ry}	”	1.5
$\alpha = S_u/S_{vb}$	%	32
S	$(\times 10^{-7}\mathrm{m}^2)$	3.72
S_{vb}	”	0.69

Table 5: *Fitted parameters in Model IIb for the average over nine wild-type stem segments. Fit based on efflux and tissue profile at 600 min (C_{d0} = 0.10 μM. S and S_{vb} are averages over measured values).*

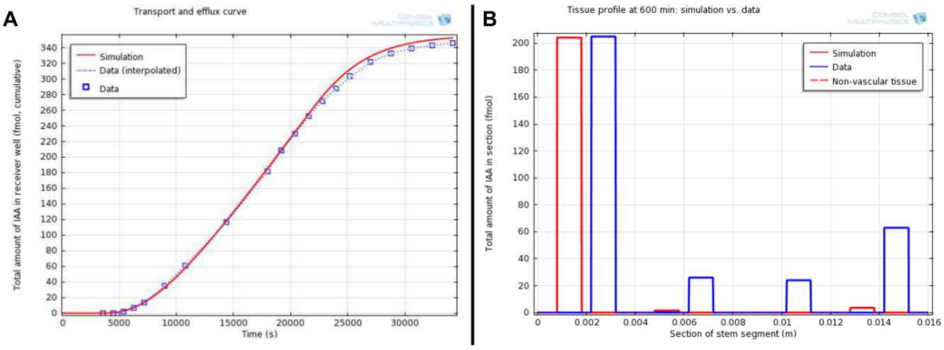

Figure 7: *Fit to Model IIb of experimental data of efflux profile of extended PAT assay (A) and tissue profile (B) at 600 min (average of nine stem segments). Corresponding parameter values are presented in Table 5.*

profile shows a good fit (Figure 7A). The stem segment almost completely empties in 600 minutes.

3.4 Model III: Combined immobilization and exchange

Both Model IIa and IIb do not fully describe all data: although efflux profiles can be fitted well with these models, issues arise either in the interpretation of parameter shift when wild-type is compared to the quadruple mutant, or the system is releasing auxin too fast. The tissue profile at 600 min of Model IIb (Figure 7B) suggested to investigate a combination of Models IIa and IIb. More auxin can then be contained within the stem segment. This yields Model III, which incorporates both immobilization of IAA and exchange to the surrounding tissue.

The equations that govern the change in time of the longitudinal density distributions of auxin for $0 < x < L$ in the u-, w-, z- and y-compartment in the stem are:

$$\partial_t u = D_u \partial_x^2 u - V \partial_x u - au + bw - \kappa_1 u + \kappa_2 z, \quad (23)$$

$$\partial_t w = D_w \partial_x^2 w + au - bw, \quad (24)$$

$$\partial_t y = D_y \partial_x^2 y, \quad (25)$$

$$\partial_t z = \kappa_1 u - \kappa_2 z. \quad (26)$$

Figure 8 shows the schematic representation of Model III.

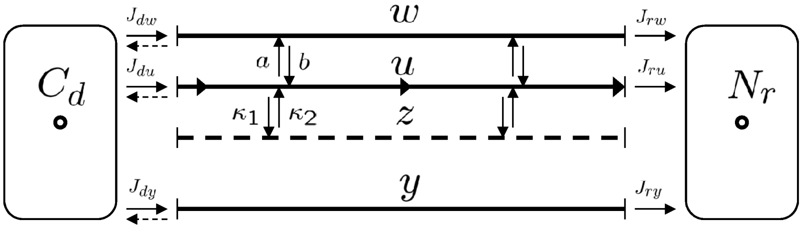

Figure 8: *Schematic representation of the 'final' model for PAT, Model III.*

The boundary conditions at $x = 0$ and $x = L$ are as in Model IIa and IIb. The dynamics of the auxin concentration in the donor well and the cumulative amount of auxin that reached the receiver well are given by

$$V_d \frac{dC_d}{dt} = -J_{du} - J_{dw} - J_{dy}. \quad (27)$$

and

$$\frac{dN_r}{dt} = J_{ru} + J_{rw} + J_{ry}. \quad (28)$$

The initial condition for (27) is $C_d(0) = C_{d0} = 1 \times 10^{-4}$ mol/m^{-3} = 0.1 μM at the start of all transport experiments, while $N_r(0) = 0$. System (23)–(26) starts with initial condition 0 for all variables. At $t = 300$ min C_d is changed to 0, thus simulating an extended PAT assay.

For reasons of symmetry in the microscopic tissue stucture, we assume that

$$P^-_{rw} = P^-_{dw}, \qquad P^-_{ry} = P^-_{dy}.$$

Fitting results:
The fitted parameter values and corresponding efflux and tissue profiles for wild-type and *aux1/lax* quadruple mutant are presented in the Main Text (Table 1 and Figure 4 for wild type; Table 2 and Figure 5 for quadruple mutant).

Discussion:
If we compare the estiamted values for the effective permeabilities p^+_a and p^-_a of the apical membrane of transport cells (see Section 2.2) with permeabilities shown in Table 1 (pooled data, Main Text), we observe that the values for P^+_{dy} and P^-_{dy} have the order of magnitude of the calculated p^+_a and p^-_a. The relatively high value of P^+_{du} (4.1×10^{-6} m/s) is probably brought about by the presence of auxin influx carriers of the AUX/LAX1-3 family and corresponds well with Rutschow *et al.* [29], who found a value of 1.5×10^{-6} m/s for the influx of auxin mediated by AUX1 using protoplasts from *Arabidopsis* roots.

Note further, that the value of the parameter P^+_{dw} (4×10^{-6} m/s) cannot be explained if we assume that import carriers are only present in transport cells of the u-compartment. Moreover, the simulations were quite insensitive to the parameter P^-_{du} (1.5×10^{-8} m/s, a sensitivity of 0.725). This insensitivity, however, can easily be explained by the strong draining of auxin from the donor boundary of the u-compartment due to strong advection.

For further discussion, see Main Text.

4 Microscopic interpretation of exchange rates

In Model IIb and III, the exchange rates for auxin from the u-compartment towards the w-compartment, a, and in opposite direction, b, are of particular interest, because they are effective transport rates that result from the underlying tissue structure in the vascular bundles and microscopic cell-to-cell

auxin transport dynamics. The fitted values for wild type and quadruple mutant plants and the changes therein for these macroscopic parameters yields insights in the microscopic dynamics as we shall now discuss.

We start with a derivation of the auxin flux between parts of a polarly auxin transporting phloem parenchyma cell and surrounding phloem tissue, or between part of a xylem parenchyma cell in a PAT channel and a neighbouring xylem vessel in the xylem, see Figure 9. The mathematical derivation and resulting expression for this flux are the same for xylem and phloem. We expect the parameter values in both cases to differ though. This local, microscopic, description of auxin flux is then up-scaled to the level of a vascular bundle with u- and w-compartment.

A thin cross section of the vascular bundle's u-compartment consists of many PAT cells, supposedly both in phloem and xylem (see Main Text, Figure 2). Similarly the w-compartment consists of various phloem cells and xylem vessels. We shall arrive at expressions for the a and b parameters in terms of the microscopic auxin transport parameters and supposed anatomical structure of the vascular bundle, in particular the distribution of PAT cells among phloem and xylem.

Auxin must travel from the cytoplasm of a cell in the u-compartment, through its cell membrane into the apoplast that connects the PAT cell and the neighbouring cell or xylem vessel. In either case, auxin in the apoplast must cross a cell membrane again to get into the cytoplasm of the neighbouring cell that is part of the w-compartment, or it must get through a vessel wall to get into a xylem vessel (cf. Figure 9). The thickness of the apoplast (ca. $1-2$ μm) in relation to the diffusivity of auxin in agar ($D_u \sim 7 \times 10^{-10}$ m^2/s, see Section 2) motivates to assume a homogeneous distribution of auxin in the apoplast in a first model for $u-w$-interaction.

Accordingly, denote by C, U and W the total *concentration* of auxin in the apoplast and a thin layer of volume V near the cell membrane both in the transporter cell and the neighbouring cell or xylem vessel. We arrive at the following system of equations for the local exchange of auxin between

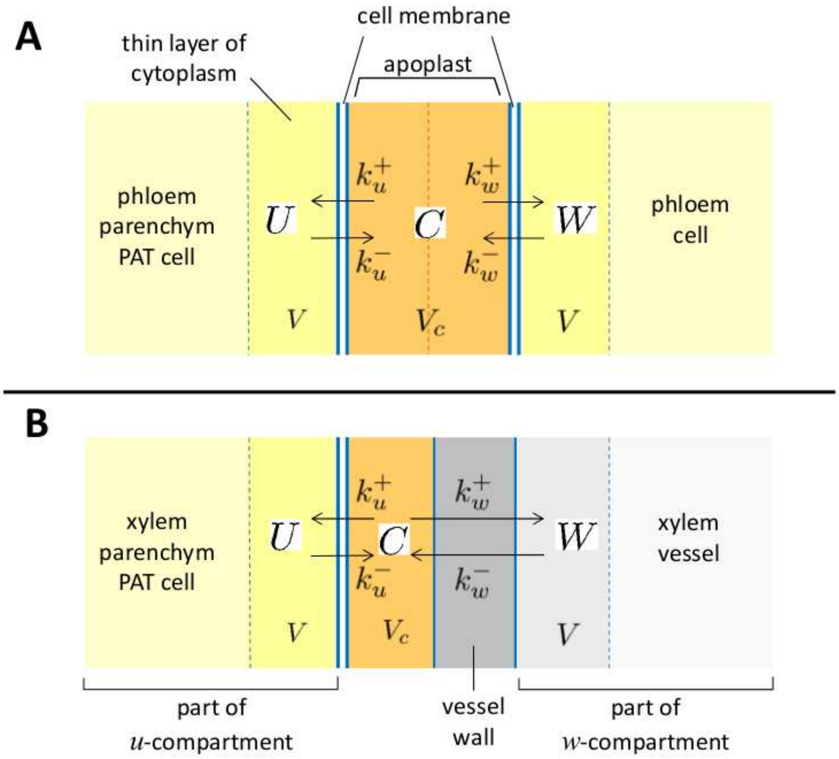

Figure 9: *Cartoon of the tissue structure in a thin cross section of the stem segment where u- and w-compartment meet at cellular level. Panel A: the assumed structure in phloem. Panel B: the assumed structure in xylem, around a xylem vessel. The cartoon is an interpretation and abstraction of expression patterns of PAT molecular markers, see Main Text, Figure 2.*

neighbouring cells or cell and xylem vessel in the u- and w-compartments:

$$\frac{dU}{dt} = k_u^+ C \cdot \frac{V_c}{V} - k_u^- U, \tag{29}$$

$$\frac{dC}{dt} = -k_u^+ C + k_u^- U \cdot \frac{V}{V_c} - k_w^+ C + k_w^- W \cdot \frac{V}{V_c}, \tag{30}$$

$$\frac{dW}{dt} = k_w^+ C \cdot \frac{V_c}{V} - k_w^- W. \tag{31}$$

Here V_c represents the volume of the thin section of apoplast. k_u^+ and k_w^+ are the (effective) rates at which auxin (in anion or protonated form) crosses the cell membrane from apoplast into u- and w-cell or xylem vessel respectively,

while k_u^- and k_w^- are the corresponding rates in opposite direction (see Figure 9).

We assume that the transport rates from apoplast into the cells are much larger than those from cell to apoplast, i.e. $k_u^-/k_u^+ \ll 1$ and similarly $k_w^-/k_w^+ \ll 1$, such that

$$\varepsilon := \frac{k_u^- + k_w^-}{k_u^+ + k_w^+} < \frac{k_u^-}{k_u^+} + \frac{k_w^-}{k_w^+} \ll 1.$$

This assumption is consistent with the *in vivo* situation where auxin in the cytoplasm is mainly in anion form (pH ≈ 7), nearly blocking transport by diffusion through the cell membrane into the apoplast, while in the acidic apoplast (pH $\sim 4-6$) it is present both in anion and protonated form. A similar reasoning can apply to the slightly acidic content of a xylem vessel (pH ≈ 6.5). Both diffusion and carrier-mediated transport (e.g. through the AUX1/LAX family of transporters) bring auxin into a cell. Transporter proteins from the PIN-family, generally assumed to realize efflux of auxin anions out of the cell, are located at the basal side of transporter cells in the stem segment, not at the lateral side.

System (29)–(31) conserves the total number of molecules

$$N_{\text{tot}} := UV + CV_c + WV.$$

So a solution to (29)–(31) starting at a point in

$$\mathcal{S}(N_0) := \{(U, C, W) \in \mathbb{R}^3 \mid N_{\text{tot}} = N_0,\ U, C, W \geq 0\}, \qquad (N_0 \geq 0),$$

will remain in $\mathcal{S}(N_0)$ for all time. Moreover, the solution converges to the unique steady state $(\bar{U}, \bar{C}, \bar{W})$ in $\mathcal{S}(N_0)$:

$$\bar{U} = \frac{k_u^+}{k_u^-}\frac{V_c}{V}\bar{C}, \quad \bar{W} = \frac{k_w^+}{k_w^-}\frac{V_c}{V}\bar{C}, \quad \bar{C} = \frac{N_0}{V_c}\left(1 + \frac{k_u^+}{k_u^-} + \frac{k_w^+}{k_w^-}\right)^{-1}.$$

We make the restriction of system (29)–(31) to $\mathcal{S}(N_0)$ non-dimensional by scaling the concentrations of U, W and C with respect to their steady state values: $\hat{U} := U/\bar{U}$, $\hat{W} := W/\bar{W}$ and $\hat{C} := C/\bar{C}$. Time is made non-dimensional by scaling with respect to the slow transport time scale $\tau_s := (k_u^- + k_w^-)^{-1}$. That is, $\hat{t} := t/\tau_s$. Then (29) and (30) become

$$\frac{d\hat{U}}{d\hat{t}} = \frac{k_u^-}{k_u^- + k_w^-}\big(\hat{C} - \hat{U}\big), \tag{32}$$

$$\varepsilon\frac{d\hat{C}}{d\hat{t}} = -\hat{C} + \hat{U} + \hat{W}. \tag{33}$$

An equation similar to (32) holds for $\hat{W}$. So a quasi-steady state assumption for $\hat{C}$ is made in first approximation. Let C^* be the corresponding state for C in dimensional form:

$$C^* = \frac{V}{V_c} \cdot \frac{k_u^- U + k_w^- W}{k_u^+ + k_w^+}. \tag{34}$$

System (29)–(31) then reduces to

$$\frac{dU}{dt} = -k^- U + k^+ W, \tag{35}$$

$$\frac{dW}{dt} = \ \ k^- U - k^+ W, \tag{36}$$

with

$$k^+ = \frac{k_u^+ k_w^-}{k_u^+ + k_w^+}, \qquad k^- = \frac{k_u^- k_w^+}{k_u^+ + k_w^+}. \tag{37}$$

The rates $k_u^\pm$ and $k_w^\pm$ can be expressed in terms of effective permeabilities $P_u^\pm$ and $P_w^\pm$ of the cell membrane or vessel wall (in terms of total auxin concentration):

$$k_u^\pm = \frac{P_u^\pm A_{loc}}{V}, \qquad k_w^\pm = \frac{P_w^\pm A_{loc}}{V},$$

where A_{loc} is the small (local) area of part of the cell membrane or vessel wall that is in contact with the intermediate apoplast in the thin cross section of the stem segment. The effective local flux *density* of auxin through the membrane-apoplast-membrane or membrane-apoplast-vessel wall system then equals

$$J_{loc} = \frac{V}{A_{loc}}(k^- U - k^+ W) = \frac{P_u^- P_w^+}{P_u^+ + P_w^+} \cdot U - \frac{P_u^+ P_w^-}{P_u^+ + P_w^+} \cdot W. \tag{38}$$

Expression (38) for the local flux density can now be used to derive an expression for the parameters a and b on the larger scale.

In a first approach, let us assume that the u-compartment is located within the xylem and that the distribution of auxin within the u- and w-compartments is homogeneous in transversal direction: near the cell membrane $U = u/S_u$ and $W = w/S_w$. Assume further that cell membranes of PAT cells (together forming the u-compartment) that connect to the w-compartment all have the same permeabilities $P_u^\pm$. We make a similar assumption for the permeabilities of the vessel walls $P_w^\pm$. Then from (38) one derives that

$$a = \frac{\Gamma}{S_u} \cdot \frac{P_u^- P_w^+}{P_u^+ + P_w^+}, \qquad b = c \cdot \frac{\Gamma}{S_u} \cdot \frac{P_u^+ P_w^-}{P_u^+ + P_w^+}, \tag{39}$$

where $c = S_u/S_w$ and Γ is length of the curve separating u- and w-compartment is a cross section of the stem segment.

As explained in Main Text, Section 5.2, comparison of the fitted values for a and b for wild type and *aux1/lax* quadruple mutant suggests that the AUX1/LAX auxin import carriers implement a 25-fold increase in membrane permeability. Further analysis of the relationship between macroscopic phenomenological parameters, like a and b, and microscopic parameters for various assumptions on anatomical structure and transport mechanisms is currently in progress.

5 Parameter optimization

5.1 Cost function and good fit

The experimental data consists of a time series of N measurements of the amount $\hat{N}_{r,i}$ of radioactively labeled auxin that accumulated in the receiver well at time $t = t_i$ $(i = 1, 2, \ldots, n)$ as measured in the efflux profile in a standard or extended PAT assay. Typically 15-18 time points were taken, non-equidistant to properly resolve the onset of steady polar auxin transport and the decrease in transport in an extended PAT experiment (see Main Text, Material & Methods). The deviation between these measurements and simulated solution $N_r(t)$ is quantified by an averaged and weighted square difference cost function

$$\gamma_{\mathrm{ep}} := \sum_{i=1}^{N} \omega_i \big(N_r(t_i) - \hat{N}_{r,i} \big)^2 \tag{40}$$

for the efflux profile. The weights satisfy $\omega_i > 0$ and $\sum_{i=1}^{N} \omega_i = 1$. They allow to put more emphasis on the curved sections of the transport profile at start or end of an extended PAT experiment. The first and last eight time points received a ten-fold higher weight than the remaining points in our ultimate parameter optimizations.

For the tissue profile a similar measure was used for the deviation between the empirically determined total amounts of auxin in each of the four 4 mm stem segments $\hat{I}_j$ $(j = 1, \ldots, 4)$ in a tissue profile and the amounts I_j determined from the simulation for the given parameter setting by numerically integrating the simulated solutions u, w, y and z at the end-time

$t = t_{\text{end}}$ of the standard or extended PAT experiment over the x-intervals $[\frac{1}{4}(j-1)L, \frac{1}{4}jL]$:

$$I_j := \int_{\frac{1}{4}(j-1)L}^{\frac{1}{4}jL} (u + w + y + z)(x, t_{\text{end}})\, dx. \tag{41}$$

The corresponding cost function is

$$\gamma_{\text{tp}} = \frac{1}{4} \sum_{j=1}^{4} \big(I_j - \hat{I}_j\big)^2. \tag{42}$$

Parameters in the model(s) are optimized by minimizing the total cost function

$$\gamma := \gamma_{\text{ep}} + \gamma_{\text{tp}}. \tag{43}$$

The optimization procedure that we describe below is stopped once the total cost $\gamma(p)$ for an obtained parameter setting p is of the order of magnitude of the square of the experimental accuracy of measuring amounts of tritium-labeled IAA. This experimental accuracy is approximately 1.5-2 fmol. Accordingly, a fit is considered good or optimal when $\gamma(p)$ is about 2 - 4 (fmol^2) or smaller.

5.2 Parameter optimization algorithm

The parameter optimization algorithm that we use is a variant of the classical Gradient Descent method (which dates back to 1847 [6]). Although the classical method does not have a very good convergence performance among optimization algorithms [1], we have chosen to use a variant, the Gradient Descent method with an inexact linear search for an appropriate step-size, because of its simplicity in implementation and computational complexity. Application of this algorithm with the ability to examine its progress stepwise would give a quick but rough impression of the cost function landscape $p \mapsto \gamma(p)$. If this would seem favourable one could decide to start using a more advanced algorithm.

Of these, the use a Newtonian method is insuitable anyhow as it requires the computation of the Hessian. This is far too expensive computationally for the number n of parameters $p_1, \ldots, p_n$ that is involved ($n = 16$ in Model III. In this case it would require $\sim n^2 = 256$ COMSOL Multiphysics runs to

approximate the Hessian). A quasi-Newtonian method might be considered instead (e.g. Broyden).

In the Gradient Descent method, a new parameter setting p^* is determined from the previous setting p by

$$p^* = p - h\nabla\gamma(p), \tag{44}$$

where

$$-\nabla\gamma(p) = -\big(\frac{\partial\gamma}{\partial p_1}(p), \ldots, \frac{\partial\gamma}{\partial p_n}(p)\big), \tag{45}$$

is the negative gradient of the cost function γ at p, which points in the direction of steepest (local) decrease of γ at p. h is a step-size that can vary per step. In the classical Gradient Descent method h is chosen to minimize γ in the direction of $-\nabla\gamma(p)$:

$$h = \arg\min_{h'>0} \gamma(p - h'\nabla\gamma(p)). \tag{46}$$

Other methods for selecting h may be followed, like the Barzilai-Borwein step-size [3]. We determine h by the following inexact linear search.

Instead of approximating the exact minimum of γ on the half line ℓ_p emanating from p in the direction of $-\nabla\gamma(p)$, we sample γ at new points p_i^* on ℓ_p, subsequently farther away from p either until (a) one of the parameter values becomes negative, (b) γ starts increasing or (c) $2n$ samples have been taken. In case (b), if γ increases in the first step already, a miminum must exists on the line ℓ_p between $p = p_0^*$ and p_1^*. In that case a point on ℓ_p between these two is sought by taking a smaller step such that γ has smaller value there than in p and p_1^*. In either subcase of (b) one obtains three points p_i^*, p_{i+1}^* and p_{i+2}^* at increasing distance from p such that

$$\gamma\big(p_i^*\big) \ > \gamma\big(p_{i+1}^*\big) \quad \text{and} \quad \gamma\big(p_{i+2}^*\big) \ > \gamma\big(p_{i+1}^*\big), \tag{47}$$

By means of the Golden Ratio method [13] we then approximate the minimum of γ that must lie either between p_i^* and p_{i+1}^* or between p_{i+1}^* and p_{i+2}^*. In this method a new point $\hat{p}^*$ is determined by

$$\hat{p}^* := p_i^* + p_{i+2}^* - p_{i+1}^*, \tag{48}$$

that lies between p_i^* and p_{i+1}^* or p_{i+1}^* and p_{i+2}^* on the line ℓ_p. The value $\gamma(\hat{p}^*)$ is determined. If $\hat{p}^*$ lies in the 'interval' from p_i^* to p_{i+1}^* and

$$\gamma\big(p_i^*\big) \ > \gamma\big(\hat{p}^*\big) \quad \text{and} \quad \gamma\big(p_{i+1}^*\big) \ > \gamma\big(\hat{p}^*\big), \tag{49}$$

then one continues with the points p_i^*, $\hat{p}^*$ and p_{i+1}^* instead of p_i^*, p_{i+1}^* and p_{i+2}^*. Otherwise one continues with $\hat{p}^*$, p_{i+1}^* and p_{i+2}^*. A similar reduction of the interval is obtained when $\hat{p}^*$ is between p_{i+1}^* and p_{i+2}^*. The Golden Ratio method chooses the spacing between the points such that the spacing between p_i^*, $\hat{p}^*$ and p_{i+1}^* and $\hat{p}^*$, p_{i+1}^* and p_{i+2}^* is similar, which yields the golden ratio that is in its name.

This approximation method is continued until either in total $2n$ steps have been made during linear search, or we obtained a 'good fit' (see definition above).

The step-size that is then chosen in the Gradient Descent method is the one among the sampled step-sizes that provided the largest decrease in γ during the linear search, but not necessarily the maximal one, as in the classical case. In this sense, the linear search is inexact.

5.3 Implementation

The parameter optimization algorithm was implemented in MATLAB R2008b (version 7.7.0.471), coupled to COMSOL Multiphysics 4.2a running as time-dependent solver of the auxin transport model through COMSOL's 'LiveLink for MATLAB'. The computations were performed on a Windows 7 (64 bit) laptop with Intel Core i7-2630 QM CPU at 2.00 GHz with 6 GB RAM with MATLAB and COMSOL running on this single machine in COMSOL Server architecture.

The weights ω_i in the efflux profile cost function γ_{ep} (40) were taken such that the first and last eight time points have a weight that is ten-fold higher than for the remaining weights that have equal value. Because the structure of Model III is such that y is not directly coupled to u, w or z, the y-related parameters D_y, $P_{dy}^{\pm}$ were initially ignored by setting them to a physiologically reasonable value. The total amount of IAA in the first 4-mm subsegment is ignored in initial fitting, as much of the accumulation found there can be attributed to accumulation in y. Thus we used a modified γ_{tp}:

$$\tilde{\gamma}_{\text{tp}} = \frac{1}{3}\sum_{j=2}^{4}\big(I_j - \hat{I}_j\big)^2, \tag{50}$$

i.e. only the last three 4-mm subsections are taken into account. Accordingly we used a modified cost function $\tilde{\gamma} := \gamma_{\text{ep}} + \tilde{\gamma}_{\text{tp}}$. Once an optimal fit for $\tilde{\gamma}$ was obtained, the original cost function γ was then optimized by tuning the

y-parameters only to fit the amount of auxin in the first 4-mm subsection in the tissue profile.

In the Gradient Descent method the gradient of the cost function at a particular parameter setting is numerically approximated using the central difference approximation for each component:

$$\nabla\gamma(p)_i \approx \frac{1}{2\Delta p_i}\big(\gamma(p+\Delta p_i\,\mathbf{e}_i) - \gamma(p-\Delta p_i\,\mathbf{e}_i)\big), \tag{51}$$

where $\mathbf{e}_i$ is the i-th standard basis vector in $\mathbb{R}^n$. As the average of the left and right difference approximation to the derivative at p it performs typically better as approximation than either of these. We take $\Delta p_i/p_i = 2\%$ for each i in the computations.

Since parameters and cost function are not dimension-free we have to modify (44). We do so by choosing a reference value $p_{i,ref}$ for each parameter, typically its physiological order of magnitude, such that all dimension free parameters $\hat{p}_i := p_i/p_{i,ref}$ have similar order of magnitude. For the cost function we take the reference value γ_{ref} equal to the square experimental accuracy in determining the amount of labeled auxin. Then we perform Gradient Descent on the minimization problem of $\hat{\gamma} := \gamma/\gamma_{ref}$ as function of the dimension free parameters $\hat{p}_i$ This amounts to dimensional parameter update step

$$p_i^* := p_i - \frac{\partial\gamma}{\partial p_i}\cdot\frac{p_{i,ref}^2}{\gamma_r ef}\cdot h \tag{52}$$

for each i.

5.4 Discussion

When examining the performance of the Gradient Descent method described above, we found when starting at randomly chosen points in parameter space, that the algorithm would often quickly converge to a parameter setting in which the total costs function obtained a seemingly suboptimal minimal value (e.g. $\gamma \approx 30$) from which it could not find a path to a better fit. Although we cannot prove rigorously that the cost function has multiple local minima, it appears that there are many distributed over parameter space. Thus we made not too large perturbations of a parameter setting that we obtained initially by fitting manually, and that provided a good fit 'by eye' in the efflux and tissue profiles. It turned out that the algorithm could recover and improve on these manual fits, although only slightly in some

cases. We report on these improved fits in the parameter tables above and in the Main Text. The manual fits contributed much to our understanding of the dynamics of the ultimate model, the dependence of simulated efflux and tissue profiles on parameters in particular.

The cost function landscape that we found gives the impression that it will be hard to perform a fully automated search of parameter space and find (all) globally optimal fits. It seems that currently available statistical techniques for parameter estimation for *time-dependent* coupled systems of partial and ordinary differential equations must be improved or extended in order to be able to cover Model III that we consider. Recent mathematical literature seems to cover mainly the time-independent case, which is technically already quite challenging mathematically [5, 9]. The development of such techniques is beyond the scope of this study.

Acknowledgment: We thank Mathisca de Gunst en Ivan Vujacic from the VU University Amsterdam, The Netherlands, for discussing feasible statistical techniques for parameter estimation for our ultimate model, Model III.

References

[1] Akaike, H. (1959). On a successive transformation of a probability distribution and its application to the analysis of the optimum gradient method, *Ann. Inst. Statist. Math. Tokyo* **11**, 1–17.

[2] Baluška, F., Šamaj, J. and D. Menzel (2003). Polar transport of auxin: carrier-mediated flux across the plasma membrane or neurotransmitter-like secretion? *TRENDS in Cell Biol.* **13**(6), 282–285.

[3] Barzilai, J. and J.M. Borwein (1988). Two-point step size gradient methods, *IMA J. Num. Anal.* **8**, 141–148.

[4] Boot K.J.M., Libbenga K.R., Hille S.C., Offringa R. and B. van Duijn (2012). Polar auxin transport: an early invention. *J. Exp. Botany* **63**, 4213–4218.

[5] Bui-Thanh, T. and M. Girolami (2014). Solving large-scale PDE-constrained Bayesian inverse problems with Riemann manifold Hamiltonian Monte Carlo.

[6] Cauchy, A. (1847), Méthode généralepour la resolution des systèms d'équations simultanées, *Comp. Rend. Sci Paris* **25**, 536–538.

[7] Delbarre, A., Muller, Ph., Imhoff, V., Morgat, J.-L., and H. Barbier-Brygoo (1994), Uptake, accumulation and metabolism of auxins in tobacco leaf protoplasts. *Planta* **195**, 159–167.

[8] Delbarre, A., Muller, Ph., Imhoff, V., and J. Guern (1996), Comparison of mechanisms controlling uptake and accumulation of 2,4-dichlorophenoxy acetic acid, naphthalene-1-acetic acid, and indole-3-acetic acid in suspension-cultured tobacco cells. *Planta* **198**, 532–541.

[9] De los Reyes, J.C. (2015). *Numerical PDE-Constrained Optimization*, Springer Briefs in Optimization, Springer.

[10] Fasano, J.M., Swanson, S.J., Blancaflor, E.B., Dowd, P.E, Kao, T.H, and S. Gilroy (2001), Changes in root cap PH are required for the gravity response of the *Arabidopsis* root, *Plant Cell* **13**, 907–921.

[11] Friml, J., and K. Palme (2002), Polar auxin transport – old questions and new concepts?, *Plant Mol. Biol.* **49**, 273–284.

[12] Goldsmith, M.H.M., Goldsmith, T.H. and M.H. Martin (1981). Mathematical analysis of the chemosmotic polar diffusion of auxin through plant tissues, *Proc. Natl. Acad. Sci. U.S.A.* **78**, 976–980.

[13] Kiefer, J. (1953). Sequential minimax search for a maximum, *Proc. Amer. Math. Soc.* **4**(3), 502-506.

[14] Kotyk, A., Janáček, K. and J. Koryta (1988). *Biophysical Chemistry of Membrane Functions.* John Wiley & Sons.

[15] Kramer, E.M. (2002). A mathematical model of pattern formation in the vascular cambium of trees, *J. Theor. Biol.* **216**, 147–158.

[16] Kramer, E.M. (2004). PIN and AUX/LAX proteins: their role in auxin accumulation, *Trends in Plant Science* **9**, 578–582.

[17] Kramer, E.M. (2006). How far can a molecule of week acid travel in the apoplast or xylem?, *Plant Physiol.* **141**, 1233–1236.

[18] Kramer, E.M. (2008). Computer models of auxin transport: a review and commentary. *J. Exp. Bot.* **59**, 45–53.

[19] Kramer, E.M. and M.J. Bennett (2006). Auxin transport: a field in flux, *Trends Plant Sci.* **11**(8), 382–386.

[20] Kramer, E.M., Rutschow, H.L. and S.S. Mabie (2011). AuxV: a database of auxin transport velocities, *Trends Plant Sci.* **16**(9), 461–462.

[21] Larsen, P. (1955). Growth substances in higher plants. In *Modern methods of plant physiology*, vol. 3 (ed. K. Paech & M.V. Tracey), p. 565. Berlin: Springer.

[22] Li, J., Yang, H., Ann Peer, W., Richter, G., Blakeslee, J., Bandyopadhyay, A., Titapiwantakun, B., Undurraga, S., Khodakovskaya, M., Richards, E.L, Krizek, B., Murphu, A.S., Gilroy, S. and R. Gaxiola (2005). *Arabidopsis* H^+-PPase AVP1 regulates auxin-mediated organ development, *Science* **310**, 121–125.

[23] Mitchison, G.J. (1980). The dynamics of auxin transport, *Proc. R. Soc. Lond. B* **209**, 489–511.

[24] Peremyslov, V.V., Morgun E.A., Kurth, E.G., Kakarova, K.S., Koonin, E.V. and V.V. Dolja (2013). Identification of myosin XI receptors in *Arabidopsis* defines a distinct class of transport vesicles, *The Plant Cell* **25**, 3022–3038.

[25] Raven, J.A. (1975). Transport of indolacetic acid in plant cells in relation to pH and electrical potential gradients, and its significance for polar IAA transport. *New Phytol.* **74**, 163–172.

[26] Raven, J.A. (2013). Polar auxin transport in relation to long-distance transport of nutrients in the Charales, *J. Exp. Botany* **64**(1), 1–9.

[27] Rubery, P.H. (1979). The effect of 2,4-dinitrophenol and chemical modifying reagents on auxin transport by suspension-cultured crown gall cells, *Planta* **144**, 173–178.

[28] Rubery, P.H. and A.R. Sheldrake (1974). Carrier-mediated auxin transport, *Planta* **118**, 101–121.

[29] Rutschow, H.L., Baskin, T.I, and E.M. Kramer (2014), The carrier AUXIN RESISTANT (AUX1) dominates auxin flux into Arabidopsis protoplasts, *New Phytologist* **204**, 536–544. (DOI: 10.1111/nph.12933).

[30] Shen, J., Zeng, Y., Zhuang, X., Sun L., Yao, X., Pimpl, P. and L. Jiang (2013). Organelle pH in the *Arabidopsis* endomembrane system, *Mol. Plant* **6**, 1419-1437.
